# Heptanuclear Mixed-Valence Co_4_^III^Co_3_^II^ Molecular Wheel—A Molecular Analogue of Layered Double Hydroxides with Single-Molecule Magnet Behavior and Electrocatalytic Activity for Hydrogen Evolution Reactions

**DOI:** 10.1021/acs.inorgchem.3c04065

**Published:** 2024-03-25

**Authors:** Biplab Biswas, Anjila I. Siddiqui, Mithun Chandra Majee, Swadhin Kumar Saha, Biswajit Mondal, Rajat Saha, Carlos J. Gómez García

**Affiliations:** † Department of Chemistry, Kazi Nazrul University, Asansol 713340, West Bengal, India; ‡ Department of Chemistry, Hooghly Mohsin College, Chinsurah 712101, West Bengal, India; § Department of Chemistry, IIT Gandhinagar, Palaj 382355, Gujarat, India; ∥ Department of Chemistry, BB College, Asansol 713303, West Bengal, India; ⊥ Departamento de Química Inorgánica, Universidad de Valencia, Burjasot, Valencia 46100, Spain

## Abstract

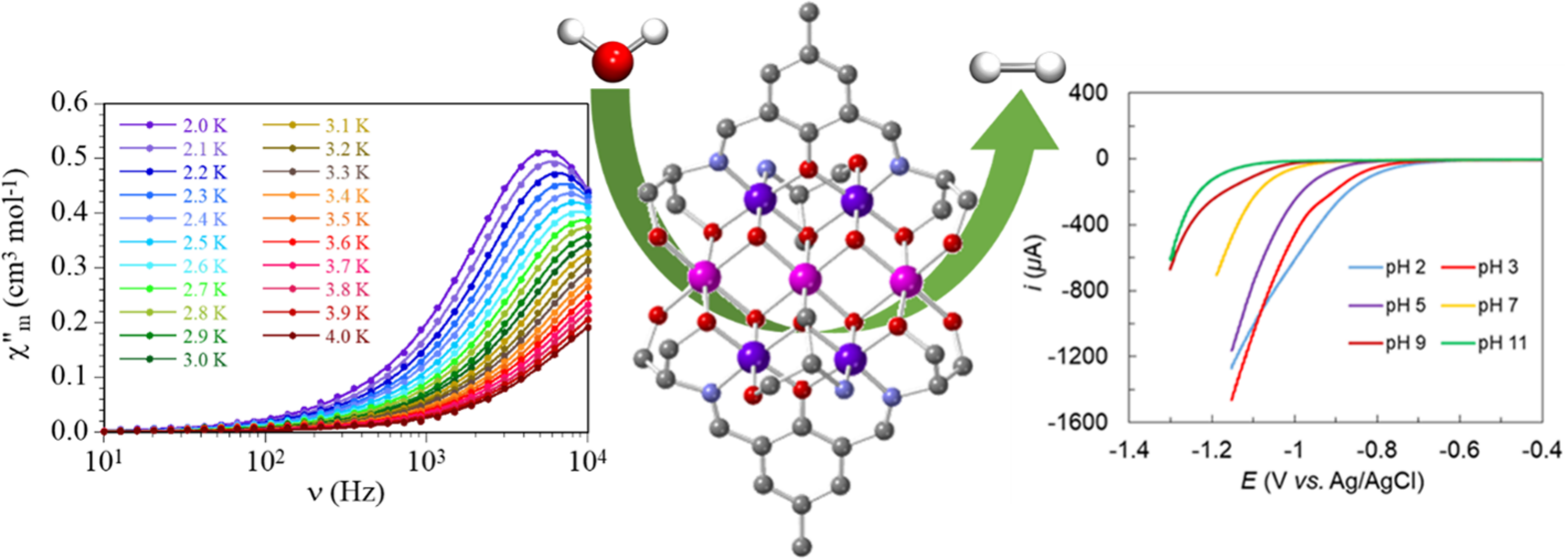

We present a bifunctional heptanuclear cobalt(II)/cobalt(III) molecular complex formulated as [Co_7_(μ_3_–OH)_4_(H_2_L^1^)_2_(HL^2^)_2_](NO_3_)_6_·6H_2_O (**1**) (where H_5_L^1^ is 2,2′-(((1E,1′E)-((2-hydroxy-5-methyl-1,3-phenylene)bis(methanylylidene))bis(azanylylidene))bis(propane-1,3-diol)) and H_2_L^2^ is 2-amino-1,3-propanediol). Compound **1** has been characterized by single-crystal X-ray diffraction analysis along with other spectral and magnetic measurements. Structural analysis indicates that **1** contains a mixed-valence Co_7_ cluster where a central Co(II) ion is connected to six different Co centers (four Co^III^ and two Co^II^ ions) by four μ_3_–OH groups, giving rise to a planar heptanuclear cluster that resembles a molecular fragment of a layered double hydroxide (LDH). Two triply deprotonated (H_2_L^1^)^3–^ ligands form the outer side of the cluster while two singly deprotonated (HL^2^)^−^ ligands are located at the top and bottom of the central heptanuclear core. Variable temperature magnetic measurements indicate the presence of weak ferromagnetic Co^II^···Co^II^ interactions (*J* = 3.53(6) cm^–1^) within the linear trinuclear Co^II^ cluster. AC susceptibility measurements show that **1** is a field-induced single-molecule magnet (SMM) with τ_0_ = 8.2(7) × 10^–7^ s and *U*_eff_ = 11.3(4) K. The electrocatalytic hydrogen evolution reaction (HER) activity of **1** in homogeneous phase shows an overpotential of 455 mV, with a Faradaic efficiency of 81% and a TOF of 8.97 × 10^4^ μmol H_2_ h^–1^ mol^–1^.

## Abstract 2

A new heptanuclear mixed-valence cobalt cluster prepared using a compartmental ligand shows weak ferromagnetic coupling, single-molecule magnet behavior, and HER activity in homogeneous phase with a moderate overpotential and high Faradaic efficiency and turnover frequency.

## 1 Introduction

The design of bi/multifunctional magnetic materials^1^ with additional properties such as electrical conductivity,^2^ porosity,^3^ luminescence,^4^ optical properties,^5^ etc. has gained much interest in order to modulate one functionality by an external magnetic field. Recently, magnetoeletrochemistry,^6^ in which the electrocatalytic properties of a magnetic material can be modulated by an external magnetic field, has emerged as a new research topic though its industrial application has not been realized yet. Initial attempts made in recent years on this topic indicate that the synergy between the magnetic and electrocatalytic properties in a material is a key criterion for different applications.^7^

Single-molecule magnets (SMMs) are a class of compounds that show slow relaxation of the magnetization on removal of a magnetizing field.^8^ Such slow relaxation arises due to the combined effect of high-spin ground state (S_T_) and uniaxial magnetic anisotropy (*D*, defined by the Hamiltonian *H* = *D* S_T_^2^), which creates an energy barrier for the magnetic spin reversal. The energy barrier Δ is calculated as |*D*| S_T_^2^ for integer spin and |*D*| (S_T_^2^ – 1/4) for half-integer spin.^9^ SMMs are envisioned as potential candidates for high-density information storage, spintronics, quantum computing, magnetic refrigeration, etc.^10^ Research on SMMs follows diverse directions including better performance, moderate operating conditions, deposition on solid substrates, high blocking temperature, and tuning the molecular magnetism by external stimuli like temperature, pressure, etc.^11^ One of the major goals is the understanding of the structure–property relationship to develop rational design principles for SMMs.^12^ The first reported SMM, [Mn_12_O_12_(OAc)_16_(H_2_O)_4_],^13^ motivated inorganic chemists to design polynuclear homo/heterometallic clusters showing SMM behavior.^14^

To date, different transition metal-based polynuclear complexes have been synthesized for the above-said purpose, but, in this regard, cobalt(II) complexes are less explored.^15^ Due to strong orbital contribution, in six or five coordinated complexes, Co^II^ (*S* = 3/2) ions show large zero-field splitting and Kramers doublets (KDs). The highly anisotropic low-lying energy Kramers levels constitute a thermal energy barrier for the reversal of magnetization, which results in SMM.^16^ Following such intriguing characteristics, the design of SMMs using polynuclear cobalt complexes has gained much attention.

Rapid depletion of fossil fuel reserves and the consequent growing energy demands as well as global warming have triggered the search for alternative sustainable energy sources.^17^ Green hydrogen is considered one of the best choices as a renewable fuel for its high-energy storage capacity, carbon-free, and environmentally benign nature.^18^ Hydrogen is also an important reagent for the reduction of different chemicals, the synthesis of ammonia, hydrocarbons, etc.^19^ Electrocatalytic reduction of aqueous protons is considered one of the most reliable strategies for sustainable production of hydrogen,^20^ and thus, electrocatalytic hydrogen evolution reaction (HER) from water has become a contemporary area of interest.^21^ Pt is considered the most efficient catalyst for HER; however, low abundance and high cost restrict its industrial applications.^22^ Therefore, the search for cheap, efficient, and non-noble metal-based robust catalysts for HER has become a key challenge in recent years.^23^ Recently, cobalt-based molecular complexes have emerged as realistic candidates for H_2_ evolution catalysts due to their intrinsic oxygen tolerance and moderate proton reduction ability.^24^ Several cobalt complexes with N-, S-, or mixed N, O-donor-based ligands have been reported in the past decade.^25^ However, the low aqueous solubility of these cobalt complexes has limited the investigations to organic media or a mixed organic–aqueous medium; thus, there is huge interest in designing water-soluble cobalt-based molecular catalysts for the direct evolution of hydrogen from water. Bren et al. have reported a water-soluble biomolecular catalyst, acylated Co-microperoxidase-11 (CoMP11-Ac), which shows electrocatalytic HER activity from water (pH = 7) with an overpotential of 850 mV, a Faradaic efficiency of 95 ± 3%, and a TON of 2.5 × 10^4^.^26^ The same authors have also performed a detailed investigation of a water-soluble cobalt(III) complex having small peptide chain as the only ligand (Gly–Gly–His) that shows electrocatalytic HER behavior with an overpotential of 600 mV (pH = 8), a Faradaic efficiency of 91 ± 3%, and a TON of 275.^27^ Detailed investigations have revealed that the stability and catalytic overpotential depend on the presence of electron donor/acceptor moieties on the axial pyridyl group attached to the square planar Co complex.^28^ Dutta and co-workers have modulated the electrocatalytic HER activity by incorporating several functional groups at the attached axial pyridyl moiety of the cobaloxime framework.^29^ Again, they have also shown that the presence of protic functionalities at the outer coordination sphere of the metal ions has a strong influence on the electrocatalytic activity.^30^ Nevertheless, the use of mixed-valence Co complexes as molecular HER catalysts is rare.^28^

In the present work, to design a bifunctional molecular material, by encompassing both single-molecule magnet behavior and electrocatalytic HER activity, we follow a strategy based on cobalt ions and a heptadentate compartmental ligand since (i) cobalt can simultaneously show interesting magnetic and catalytic properties, (ii) cobalt(II), when coordinated to moderate or strong field ligands, can be easily oxidized to cobalt(III) and can form mixed-valence (II/III) compounds, highly sought-after in electrocatalysis, and (iii) the use of a heptadentate compartmental ligand that can bind to more than one metal center is expected to form a large molecular cluster. Following this strategy, by using a mixed N,O-donor polydentate ligand, we have prepared a heptanuclear mixed-valence cobalt(II/III) compound formulated as [Co_7_(μ_3_–OH)_4_(H_2_L^1^)_2_(HL^2^)_2_](NO_3_)_6_·6H_2_O (**1**) (where H_5_L^1^ is 2,2′-(((1E,1′E)-((2-hydroxy-5-methyl-1,3-phenylene)-bis(methanylylidene))-bis(azanylylidene))-bis(propane-1,3-diol)) and H_2_L^2^ is 2-amino-1,3-propanediol). Compound **1** has been characterized by single-crystal X-ray crystallography, spectroscopic techniques, magnetic measurements, and electrocatalytic HER activity studies. Structural analysis shows that **1** is a heptanuclear molecular wheel that contains three Co^II^ and four Co^III^ ions connected by four internal μ_3_–OH bridges. Compound **1** shows field-induced SMM behavior and electrocatalytic HER activity with an onset potential value of −0.77 V with a Faradaic efficiency of 81% and a TOF of 8.97 × 10^4^ μmol H_2_ h^–1^ mol^–1^.

## 2 Experimental Section

### 2.1 Materials and Methods

Cobalt(II) nitrate hexahydrate and 2-amino-1,3-propanediol (**H**_**2**_**L**^**2**^; 98%) were purchased from Aldrich and used as received. 2-Hydroxy-5-methylisophthalaldehyde was synthesized by using the procedure described in the literature.^31^ All other chemicals were AR grade and were used as received, without further purification. Elemental analysis (C, H, and N) was carried out using a PerkinElmer 240C elemental analyzer. Fourier transform infrared (FT-IR) spectra were recorded with a Nicolet Impact 410 spectrometer with KBr pellets in the range of 400–4000 cm^–1^. Absorption spectra were recorded on a SHIMADZU UV 1800 spectrometer in solution. ^1^H NMR spectra were recorded on 400 MHz Bruker NMR spectrometers using TMS as the internal standard. Chemical shifts are reported in parts per million (ppm). When peak multiplicities are given, the following abbreviations are used: s, singlet; br s, broad singlet; d, doublet; t, triplet; and m, multiplet. Powder XRD patterns were recorded by using Cu–Kα radiation (Bruker D8; 40 kV, 40 mA). The morphology of the samples was characterized by field emission scanning electron microscopy (FE-SEM, Carl Zeiss, Germany) at 5 kV. X-ray photoelectron spectroscopy (XPS) was performed on a SCIENTA, R-3000 Analyzer using a monochromatic Al Kα source (*h*ν = 1486.6 eV). The typical vacuum in the analysis chamber during the measurements was in the range of 1 × 10^–10^ Torr. Charge neutralization was used for all measurements by using a combination of low-energy Ar^+^ ions and electrons.

### 2.2 Synthesis of the Ligand H_5_L^1^ [C_15_H_22_N_2_O_5_]

A methanolic solution (5 mL) of 2-hydroxy-5-methylisophthalaldehyde (164 mg, 1 mmol) was added to a methanolic solution (10 mL) of serinol (182 mg, 2 mmol), and the resulting solution was stirred for 3 h. The orange–yellow solid that precipitated was separated through filtration and characterized by IR and CHN analyses. Yield: 90%. Anal. Calc for C_15_H_22_N_2_O_5_: C, 58.05; H, 7.15; N, 9.03%. Found: C, 58.1; H, 7.35; N, 9.05%. IR (KBr, cm^–1^): 3355, 3137, 2940, 2833, 1644, 1528, 1420, 1385, 1244, 1228, 1080. ^1^H NMR (400 MHz, DMSO-*d*_6_) 8.55 (2H, s), 7.53 (2H, s), 4.65 (4H, brs), 3.63 (8H, m), 3.45 (2H, m), 2.26 (3H, s). A signal corresponding to phenolic OH was not seen due to peak broadening.

### 2.3 Synthesis of the Complex [Co_7_(μ_3_–OH)_4_(H_2_L^1^)_2_(HL^2^)_2_](NO_3_)_6_·6H_2_O (**1**)

A methanolic solution (5 mL) of Co(NO_3_)_2_.6H_2_O (873.4 mg, 3 mmol) was added to a methanolic solution (10 mL) of the ligand H_5_L^1^ (310.2 mg, 1 mmol), and the resultant mixture was refluxed for 12 h at 65 °C. The red solution obtained was filtered and kept for crystallization. After 2 weeks, rod-like deep red-colored X-ray quality single crystals (Figure S1) were collected. Yield: 40%. Anal. Calc. for C_36_H_70_Co_7_N_12_O_42_: C, 24.63; H, 4.03; N, 9.57%. Found: C, 24.60; H, 4.00; N, 9.60%. IR (KBr, cm^–1^): 3215, 1633, 1552, 1375, 1315, 1260, 1019.

### 2.4 Single-Crystal X-Ray Data Collection and Refinement

A single crystal of compound **1** was mounted on a Bruker SMART diffractometer equipped with a graphite monochromator and Mo Kα radiation (λ = 0.71073 Å). Unit cell parameters were determined by using the APEX2^32^ program. Data reduction was carried out by the SAINT^32^ program, and correction of absorption was performed using the SADABS^32^ program. The structure was solved using the Patterson method with SHELXS-2018/3^33^ embedded in WINGX software package^34^ and refined using SHELXL-2018/3.^35^ Subsequent difference Fourier synthesis and least-squares refinement revealed the positions of the remaining non-hydrogen atoms. Non-hydrogen atoms were refined with independent anisotropic displacement parameters. Hydrogen atoms were placed in idealized positions and refined using a riding model, with their displacement parameters fixed to be 1.2 times larger than those of the attached non-hydrogen atom. All Figures were drawn using PLATON^36^ and ORTEP.^37^ Data collection and structure refinement parameters and crystallographic data for compound **1** are given in Table 1.

**Table 1:** Crystallographic Data Collection and Refinement Parameters of Compound **1**

formula	C_36_H_70_Co_7_N_12_O_42_
formula weight	1755.55
crystal system	triclinic
space group	*P*-1
*a* (Å)	11.483(6)
*b* (Å)	11.602(5)
*c* (Å)	13.269(3)
α (°)	114.536(19)
β (°)	104.80(3)
γ (°)	99.03(2)
V (Å^3^)	1483.6(12)
*Z*	1
ρ_calc_ (g/cm^3^)	1.965
μ (Mo Kα) (mm)	2.031
*F*(000)	895
crystal size (mm^3^)	0.12 × 0.16 × 0.20
temperature, *T* (K)	296
θ_min–max_ (°)	2.1 and 27.2
total data	20488
unique data	6155
*R*_int_	0.092
observed data [*I* > 2.0 σ(*I*)]	3744
*N*_ref_	6155
*N*_par_	440
*R*	0.0821
*wR*_2_	0.1512
*S*	1.02

### 2.5 Magnetic Measurements

Variable temperature magnetic susceptibility measurements were carried out in the temperature range 2–300 K with an applied magnetic field of 0.1 T (1000 Oe) on a polycrystalline sample with a mass of 11.038 mg using a Quantum Design MPMS-XL-5 SQUID magnetometer. Isothermal magnetizations were performed at 1.9 and 5.0 K on the same sample with magnetic fields of up to 5 T. AC susceptibility measurements were performed on the same sample at 2.0 K with different applied dc fields (in the range 0–500 mT) with a field of 8 Oe oscillating in the range 10–10000 Hz using a Quantum Design PPMS-9 equipment. From the plot of the relaxation time vs the DC field (see below), we determined an optimum DC field of 200 mT (2000 Oe). Accordingly, we performed the frequency sweep from 10 to 10000 Hz at different temperatures in the range 2–4 K with an applied DC field of 200 mT. The susceptibility data were corrected for the sample holder previously measured using the same conditions and for the diamagnetic contribution of the sample as deduced by using Pascal’s constant tables.^38^

### 2.6 HER Activity Measurements

The electrochemical study was performed using a Metrohm PGSTAT 101 Autolab with a three-electrode system. Ag/AgCl (saturated KCl) and Pt wire were used as reference and counter electrodes, respectively, and the scan rate was kept at 50 mV/s. A glassy carbon electrode was used as the working electrode. The catalyst (0.5 mM) was dissolved in 100 mM phosphate buffer containing 100 mM NaCl as a supporting electrolyte at pH = 2. The pH was gradually increased to 3, 5, 7, 9, and 11 by adding the required amounts of a NaOH solution. The pH was measured with an Oakton pH 700 pH-meter.

## 3 Results and Discussion

### 3.1 Synthesis and Characterization of [Co_7_(μ_3_–OH)_4_(H_2_L^1^)_2_(HL^2^)_2_](NO_3_)_6_·6H_2_O (**1**)

**Scheme 1:**
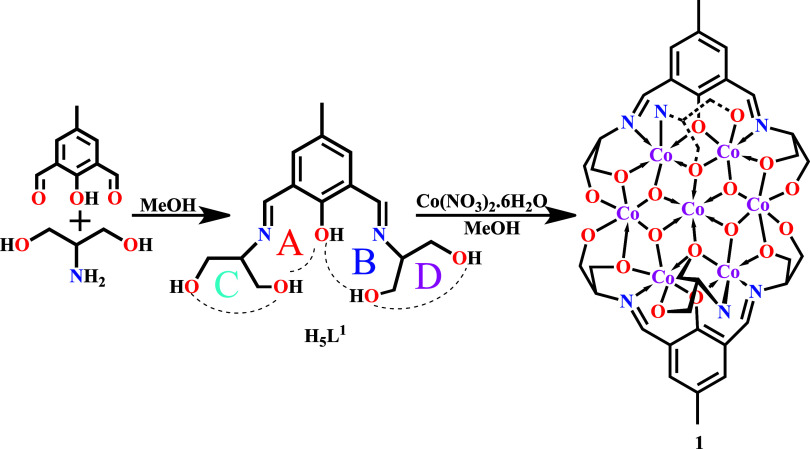
Synthesis of the Ligand H_5_L^1^ and Compound **1**

Condensation of 2-hydroxy-5-methylisophthalaldehyde and 2-amino-1,3-propanediol in methanol afforded an orange–yellow Schiff base, **H**_**5**_**L**^**1**^ (Scheme 1). This heptadentate ligand contains four different compartments to bind multiple metal centers (A–D in Scheme 1). The reaction between Co(NO_3_)_2_.6H_2_O and **H**_**5**_**L**^**1**^ in a 3:1 ratio resulted in the tetra μ_3_–OH bridged heptanuclear cobalt complex **1** with the concomitant decomposition of some **H**_**5**_**L**^**1**^ ligands to serinol moieties, which also act as bridges between Co centers within the heptanuclear cluster (Scheme 1).

The IR spectrum of the ligand H_5_L^1^ (Figure S2) shows a peak at 1644 cm^–1^ due to the azomethine (C=N) group. The coordination of the ligand H_5_L^1^ shifts the peak at lower wavenumbers. Thus, the azomethine group appears at 1633 cm^–1^ in compound **1** (Figure S3), confirming the coordination of the azomethine group to the metal centers. Compound **1** also shows a broad band centered at 3215 cm^–1^ attributed to the μ_3_-bridging O–H, the O–H bands of the lattice water, the −NH_2_ and −OH groups of the tridentate serinol ligand, as well as the −OH groups of the ligand H_2_L^2^. The ligand H_5_L^1^ has also been characterized by ^1^H NMR spectroscopy (Figure S4).

The UV–Vis absorption spectra of H_5_L^1^ and compound **1** were collected in methanolic solution in the range of 200–800 nm (Figures S5 and S6). The absorption spectrum of H_5_L^1^ shows bands at 448, 348, and 243 nm, attributed to n → π* and π → π* transitions involving free phenolic and alcoholic −OH groups and free imine groups.^39^ In compound **1**, the low intensity and lower energy bands at 526 and 404 nm are due to the d–d transition of octahedrally coordinated cobalt centers. The broad band at 404 nm may be due to the overlap of the n → π* ligand band with the d–d transitions. The other expected transitions could not be observed since, in the high-energy region, these bands overlap with the charge transfer bands. The phase purity of **1** has also been confirmed with the X-ray powder diffraction analysis (Figure S7). The SEM micrographs of a microcrystalline sample of compound **1** show flake-like crystals that agglomerate to form flower-like structures (Figure S8). XPS clearly shows the presence of both Co^II^ and Co^III^ within **1** (Figure S9).

### 3.2 Crystal Structure of [Co_7_(μ_3_–OH)_4_(H_2_L^1^)_2_(H_1_L^2^)_2_](NO_3_)_6_·6H_2_O (**1**)

**Figure 1:**
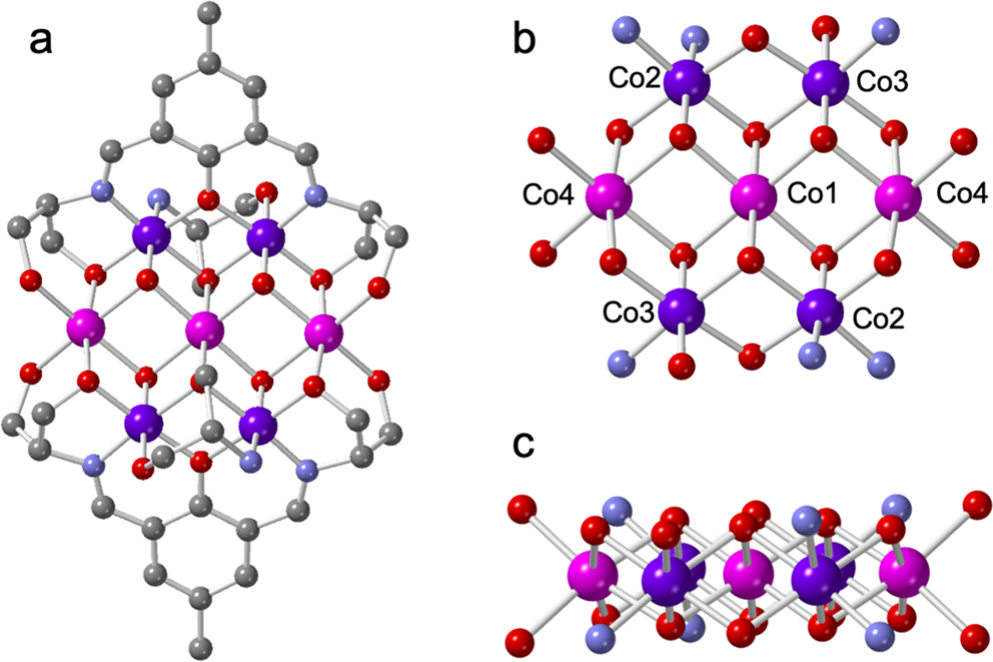
(a) View of the heptanuclear complex in **1** (H atoms are omitted for clarity). (b) Inner heptanuclear core of the complex in **1**.(c) Side view of the central Co_7_ cluster (Color code: C = Gray, O = Red, N = Blue, Co^II^ = pink, and Co^III^ = violet).

Compound **1** crystallizes in the triclinic space group *P*-1. The asymmetric unit is formed by one (H_2_L^1^)^3–^ ligand, one deprotonated serinol ligand (HL^2^)^−^ (CH_2_OH–CH(NH_2_)–CH_2_O)^−^, two μ^3^-OH^–^ groups, four cobalt atoms (Co1–Co4, with Co1 located on an inversion center), three noncoordinated NO_3_^–^ anions, and three crystallization water molecules (Figure S10). The application of the inversion center gives rise to the formula [Co_7_(μ_3_–OH)_4_(H_2_L^1^)_2_(HL^2^)_2_](NO_3_)_6_·6H_2_O observed for **1**. The bond valence sum (BVS, see below) calculations indicate that the oxidation state of Co1 and Co4 is +2, whereas it is +3 for Co2 and Co3.^40^ Since Co1 is located on an inversion center, the cluster contains four Co^III^ ions (two Co2 and two Co3) and three Co^II^ ions (two Co4 and one Co1). The complex can be described as a wheel-shaped cluster formed by mixed-valence Co_3_^II^Co_4_^III^ units, formulated as [Co_3_^II^Co_4_^III^(H_2_L^1^)_2_(HL^2^)_2_(μ_3_–OH)_4_]^6+^ (Figure 1a). The total positive charge of the seven cobalt ions in the cluster is +18 (three Co^II^ and four Co^III^), whereas the anionic charge is −12 (two (H_2_L^1^)^−3^ plus two (HL^2^)^−^ plus four (μ_3_–OH)^−^), resulting in a total cationic charge for the cluster of +6, that is balanced by six NO_3_^–^ anions.

In the mixed-valence Co_3_^II^Co_4_^III^ units, Co1 occupies the inversion center at the center of the wheel, whereas Co2, Co3, and Co4 (and their centrosymmetric equivalents) are located on the rim, connected by four μ_3_–OH^–^ groups to the central Co1 center (Figure 1b).

The seven cobalt ions present slightly distorted octahedral coordination environments. The Co–N/O bond distances around the two Co^III^ centers (Co2 and Co3) are in the range 1.856(5)–1.976(4) Å, whereas for the two Co^II^ centers (Co1 and Co4), these distances are longer as expected (in the range 2.032(4) to 2.128(4) Å).

The two Co^II^ centers (Co1 and Co4) present a CoO_6_ coordination environment (Figure S11). In Co1, it is formed by four O atoms (O8, O8′, and O9 and O9′) from the four μ_3_–OH^–^ groups and two O atoms (O6 and O6′) from two symmetry-related (HL^2^)^−^ ligands. The Co4 center is coordinated by four O atoms (O2, O3, O4′ and O5′) from two (H_2_L^1^)^3–^ ligands and by two O atoms (O8′ and O9) from two μ_3_–OH^–^ groups.

As can be seen in Figure S11, the two Co^III^ centers (Co2 and Co3) present different coordination environments: Co2 has a CoN_2_O_4_ coordination environment formed by two N-donor atoms (N2 and N3) from (H_2_L^1^)^3–^ and (HL^2^)^−^ ligands, respectively, and by four oxygen atoms: two (O1 and O5) from an (H_2_L^1^)^3–^ ligand, one (O6) from an (HL^2^)^−^ ligand, and one (O8) from a μ_3_–OH^–^ group. In contrast, Co3 has a CoNO_5_ coordination environment formed by an N atom (N1) from an (H_2_L^1^)^3–^ ligand and five oxygen atoms: two (O1 and O3) from an (H_2_L^1^)^3–^ ligand, one (O7) from an (HL^2^)^−^ ligand, and two (O6 and O9) from two μ_3_–OH^–^ groups.

The cohesion of the Co_7_ cluster is assured not only by the four central μ_3_–OH^–^ groups that connect all the cobalt centers, but also by the two different ligands (H_2_L^1^)^3–^ and (HL^2^)^−^. (H_2_L^1^)^3–^ coordinates to the four cobalt ions through two N and five O atoms, whereas (HL_2_)^−^ coordinates to the Co1, Co2, and Co3 centers through one N and two O atoms (Figure S12).

Selected coordination bond lengths and bond angles are given in Tables S1 and S2, respectively. To assign the oxidation states of the four cobalt centers, we carried out BVS calculations. The BVS values obtained are 3.47 and 3.25 for Co2 and Co3, respectively, and 2.02 and 1.87 for Co1 and Co4, respectively. These values support the formulation of the cluster in compound **1** as [Co_3_^II^Co_4_^III^(H_2_L^1^)_2_(HL^2^)_2_(OH)_4_]^6+^. These cationic clusters are connected by hydrogen bonds involving the crystallization water molecules and the nitrate anions with the amino and hydroxymethyl groups of the cationic units (Table S3). These interactions lead to the formation of chains along the *c*-axis, formed by the cationic units with a Co1···Co1 distance of 13.269 Å, which is much longer than the average distance between the central Co1 center and the peripheral Co2–Co4 centers inside the cluster (3.041 Å). The seven Co centers in the cluster are almost perfectly planar, as can be seen in Figure 1c, with a mean square root deviation of 0.00007 Å^2^.

The Co_7_ cluster is planar and formed by edge-sharing octahedra, showing a close structural resemblance with layered double hydroxides (LDHs) (Figure S13). In recent years, LDHs have gained much attention for their many applications in electrocatalysis, magnetism, water purifications, and for the fine-tuning of their properties through judicious selection of the metal ions.^41,42^ In LDHs, the metallic layer is sandwiched between two different hydroxide layers and charge-compensating anions along with guest water molecules are located in between such layers.^43^ Within the layers, the hydroxide anions connect three different metal centers, and thus, LDHs show strong magnetic coupling. Different research groups have studied the magnetic properties of such LDHs and established that the magnetic properties of LDHs depend on their composition.^41,42^ On the other hand, due to easy synthesis, low cost, high stability, compositional versatility, and high catalytic activity, LDHs have been utilized in electrocatalysis for energy storage and conversion.^44^ Herein, the metallic layer of Co_4_^III^Co_3_^II^ is sandwiched between hydroxide layers, and the cobalt ions present an edge-sharing octahedral coordination geometry. Based on such structural similarity, besides the magnetic properties, we have also studied electrocatalytic behavior of the Co_4_^III^Co_3_^II^ cluster (see below).

### 3.3 Magnetic Properties of **1**

**Figure 2:**
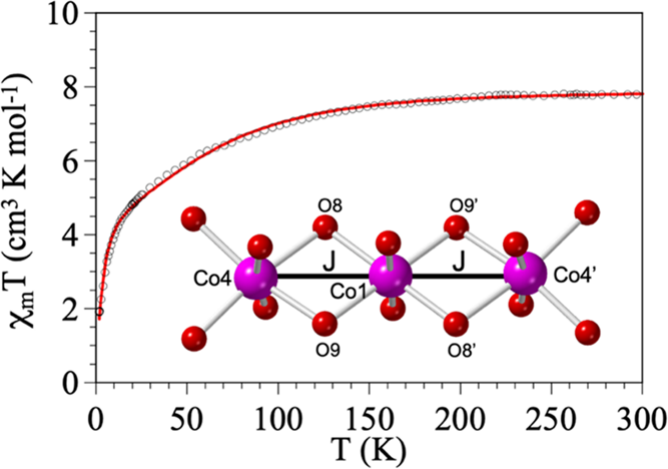
Thermal variation of χ_m_*T* for compound **1**. A solid line is the best fit to the model (see the text). The inset shows the Co_3_ cluster with the exchange coupling scheme.

The product of the molar magnetic susceptibility per Co_7_ cluster times the temperature (χ_m_*T*) for **1** shows, at room temperature, a value close to 8 cm^3^ K mol^–1^, which is the expected one for three independent Co^II^ centers in an octahedral environment with an orbital contribution.^45^ This value agrees with the presence of three Co^II^ and four diamagnetic Co^III^ centers. When the temperature is decreased, the χ_m_*T* value shows a continuous decrease to reach a value close to 2 cm^3^ K mol^–1^ at 2 K (Figure 2). This behavior indicates that the three Co^II^ ions present a spin–orbit coupling and a possible weak magnetic exchange through double hydroxido bridges.

The isothermal magnetization per formula unit (i.e., three Co^II^ ions) at 1.9 K shows a value at 5 T of almost 7 μ_B_ and points to a saturation value close to 8 μ_B_ for higher fields (Figure S15). This value suggests the presence of a large magnetic anisotropy. The values at 1.9 K are below those at 5 K, indicative of the presence of zero-field splitting at very low temperatures, in agreement with the fit of the thermal variation of the χ_m_T product.

The plot of the thermal variation of the zero-field-cooled (ZFC) and field-cooled (FC) magnetic susceptibility shows identical behaviors for both measurements (Figure S16), indicative of the absence of a long-range magnetic order at low temperatures, in agreement with the absence of an out-of-phase AC signal with zero applied DC field.

Since the structure of **1** shows that the three Co^II^ centers are connected through identical symmetry-related double μ_3_–OH^–^ groups, we have fit the magnetic properties of compound **1** with a simple symmetric linear Co^II^ trimer and two identical coupling constants (*J*), including a spin–orbit coupling (λ), an orbital reduction parameter (σ), and a zero-field splitting (ZFS) in the Co^II^ centers (*D*) using the program PHI.^46^ This model reproduces very satisfactorily the magnetic data of compound **1** in the whole temperature range with the following parameters: *J* = +3.53(6) cm^–1^, σ = 0.70(3), λ = 170(1) cm^–1^, and |*D*| = 11.8(7) cm^–1^ (with a Hamiltonian of the type −*J*(S_1_S_2_ + S_2_S_3_), solid line in Figures 2 and S14). Additionally, we have included a temperature-independent paramagnetism (Nα = 2.37(3) × 10^–3^ cm^3^ mol^–1^), to account for the contribution of the Co^III^ centers. The observed *J* value is close to the expected value for a double hydroxide bridge with Co–O–Co bond angles of 95.21(15)° and 97.22(16)°, where the coupling is expected to be ferromagnetic for Co–O–Co bond angles close to 90°.^47^ In compound **1**, the Co–O–Co bond angles are around 95–97°, and, accordingly, the J value is expected to be weak but ferromagnetic. Note that this weak ferromagnetic coupling does not result in an increase of χ_m_*T* with decreasing the temperature since it is weak and cannot overcome the decrease in χ_m_*T* due to the spin–orbit coupling and ZFS.

**Figure 3:**
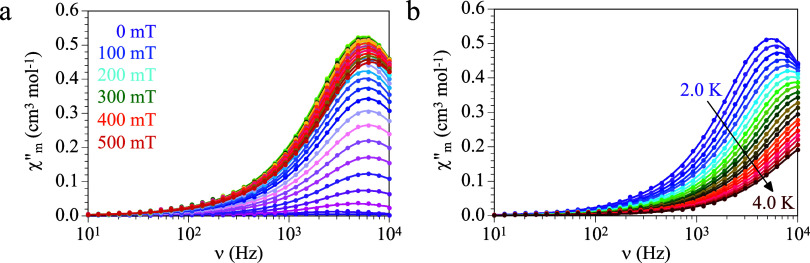
(a) Frequency dependence of χ”_m_ at 2.0 K with different applied DC fields. (b) Frequency dependence of χ”_m_ with an applied DC field of 200 mT at different temperatures for compound **1**. Solid lines are the best fit to the Debye model.

The ferromagnetic coupling found in compound **1** leads to a high-spin ground state and, therefore, points to the possible existence of a slow relaxation of the magnetization in compound **1**. Accordingly, we performed AC susceptibility measurements at low temperatures with different applied DC fields and different AC frequencies. These measurements show the presence of an out-of-phase signal (χ”_m_) only when a DC field is applied (Figure 3a). This out-of-phase signal shows a frequency dependence with a maximum that shifts to lower frequencies as the DC field increases and reaches a minimum frequency for DC fields around 200 mT. For higher DC fields, the χ”_m_ signal shifts to higher frequencies (Figure 3a). The fit of the frequency dependence of χ”_m_ to the Debye model gives the corresponding relaxation times for different DC fields. As can be seen in Figure S17, the relaxation time increases as the DC field increases and reaches a maximum value at around 200 mT. The relaxation time follows the expected variation with the DC field (eq 1)^48^ with the following parameters: *n* = 0.42(14), *A* = 2.9(3) s^–1^ mT^–*n*^, *B*_1_ = 2.9(4) × 10^4^ s^–1^, *B*_2_ = 6.8(5) × 10^–5^ mT^–2^, and *D* = 3.7(1) × 10^2^ s^–1^ (solid line in Figure S17).
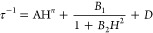
1Since the maximum relaxation time is observed for a DC field of around 200 mT, we have studied the thermal dependence of the relaxation time for compound **1** with an applied DC of 200 mT (Figure 3b).

**Figure 4:**
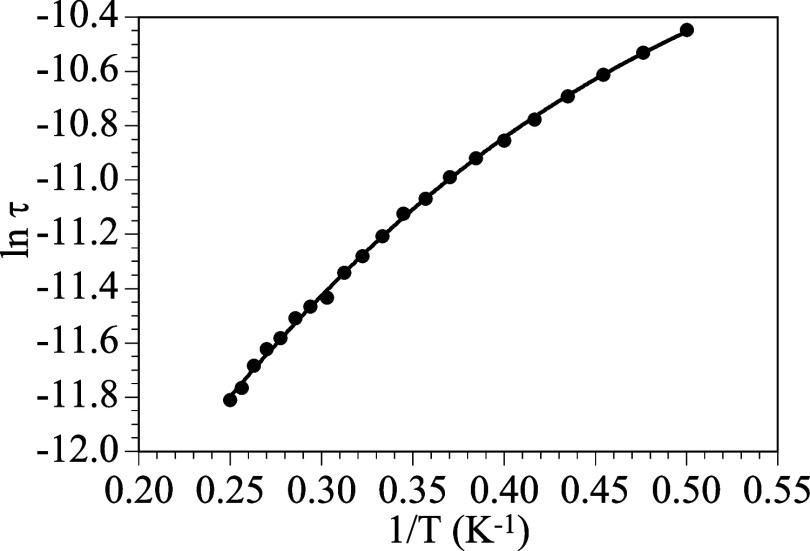
Arrhenius plot of the relaxation times with a DC field of 200 mT for **1**. The solid line is the best fit to eq 2 with the direct and Orbach terms (see the text).

These measurements show a frequency-dependent χ”_m_ signal with a maximum that moves to higher frequencies as the temperature increases. The relaxation time at each temperature has been determined with the fit of the frequency dependence of χ”_m_ to the Debye model. The Arrhenius plot (ln τ vs 1/*T*, Figure 4) shows a linear behavior at high temperatures and a slight curvature at low temperatures. This behavior indicates that the relaxation of the magnetization in compound **1** follows a thermally activated mechanism at high temperatures (Orbach mechanism, second term in eq 2). Albeit, in order to reproduce satisfactorily the behavior at low temperatures, we need to include the direct (D) mechanism (first term in eq 2).^48^
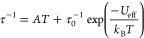
2Thus, the fit of the thermal dependence of the relaxation times to a model including direct and Raman mechanisms (eq 2) reproduces the experimental data with the following parameters: *A* = 1.52(3) × 10^4^ s^–1^ K^–1^, τ_0_ = 8.2(7) × 10^–7^ s, and *U*_eff_ = 11.3(4) K (solid line in Figure 4).

A search in the CCDC database shows that there are around 170 Co_7_ complexes of any kind reported to date. Among them, there are 47 wheel-shaped complexes with any type of double OR or OH bridges connecting the cobalt centers, as observed in compound **1** (Table 2). The most common oxidation state of the cobalt centers is Co_7_^II^ (23 examples reported), although there are also known examples of the type Co_4_^II^Co_3_^III^ (16 examples), Co_6_^II^Co^III^ (6 examples), and even Co_5_^II^Co_2_^III^ and Co_3_^II^Co_4_^III^ (with one example of each, Table 2). Compound **1** is, therefore, the second-reported wheel-shaped Co_7_ cluster with a Co_3_^II^Co_4_^III^ core.

**Table 2:** Magnetic Data of All the Reported Co_7_ Clusters with a Wheel-Shaped Structure with Any Type of Double OR Bridges Connecting the Cobalt Centers

#	CCDC	Co^II^	Co^III^	J (cm^–1^)	SMM	*U*_eff_ (K)	ref
1	AGACIV	4	3	2.6(1)	no		(49)
2	AKUZOX	7	0	>0	no		(50)
3	ALACIB	7	0	<0	yes		(50)
4	ANIDIL	7	0				(51)
5	DALCUQ	7	0	*J*_xy_–23.0(2) *J*_z_ = 14.8(2)	no		(52)
6	DIZZAP	4	3	7.4(2)	yes		(53)
7	DODWEA	7	0				(54)
8	DODWEA	6	1				(54)
9	FIVLUU	6	1				(55)
10	GIGYON	7	0	6.4(1)			(56)
11	HALFIM	7	0	>0	yes	18.8(18)	(57)
12	HOMQOS	7	0	>0	no		(58)
13	ICICEE	7	0	>0	no		(59)
14	ISAWOQ	7	0	>0	yes		(60)
15	ISORIU	4	3				(61)
16	ISOROA	4	3				(62)
17	ISUCOR	4	3				(62)
18	JILWUX	4	3	>0	yes		(62)
19	KODQEB	7	0	>0			(63)
20	KOFQUT	7	0	>0	no		(64)
21	OBOLUQ	7	0				(65)
22	OCITIF	6	1	>0	yes		(66)
23	OZOXUY	7	0				(67)
24	PORQUK	7	0	>0			(68)
25	QUNVIG	7	0				(69)
26	SADLUG	7	0	>0	no		(70)
27	SADMAN	7	0	>0	no		(70,71)
28	SOGYIY	7	0	<0			(72)
29	SOKHIK	3	4	1.5	no		(73)
30	TATYAR	5	2				(74)
31	UJOVUL	7	0	>0	no		(75)
32	UTEGIL	7	0	>0	no		(39)
33	VEPQOX	6	1	*J*_xy_ = 13/7.8 *J*_z_ = 20/12	no		(76)
34	VUJVAY	7	0	>0	no		(77)
35	XEWYEG	4	3				(78)
36	XEWYIK	4	3				(78)
37	XEWYOQ	4	3				(78)
38	XEWYUW	4	3				(78)
39	XEWZAD	4	3				(78)
40	YAJRAE	6	1		no		(76)
41	ZAYFIQ	7	0	>0			(79)
42	ZAYGAJ	6	1	<0			(79)
43	ZEGJED	4	3	>0	No		(80)
44	ZUWSUI	4	3				(81)
45	ZUWTAP	4	3				(81)
46	ZUWTET	4	3				(81)
47	ZUWTIX	4	3				(81)
48	1	3	4	3.53(6)	Yes	11.3(4)	This work

Unfortunately, the magnetic characterization has only been performed on 26 of these clusters, and among them, only in five cases a model was used to fit the magnetic exchange through the double OR bridges. In these five compounds, the coupling was found to be weak and ferromagnetic, as in compound **1**. Furthermore, the qualitative analysis of the magnetic properties performed in many of the reported Co_7_ wheel-shaped clusters shows that most of them present weak ferromagnetic couplings with Co–O–Co angles in the range 90–98°, as observed in compound **1**. In summary, the weak ferromagnetic coupling found in **1** agrees well with the observed trend in this kind of clusters.

Interestingly, among these 47 clusters, only six show single-molecule magnet (SMM) behavior at very low temperatures (three Co_7_^II^, one Co_6_^II^Co^III^ cluster, and two Co_4_^II^Co_3_^III^ clusters). Compound **1** is, therefore, the first Co_3_^II^Co_4_^III^ cluster of this type, showing SMM behavior. As can be seen in Table 2, only in one case (a Co_7_^II^ cluster), the full characterization of the relaxation process was performed in order to determine the energy barrier for the thermally activated process (Orbach mechanism). Compound **1** is, therefore, the first mixed-valence characterized SMM Co_7_ cluster.

### 3.4 Electrochemical HER Activity

**Figure 5:**
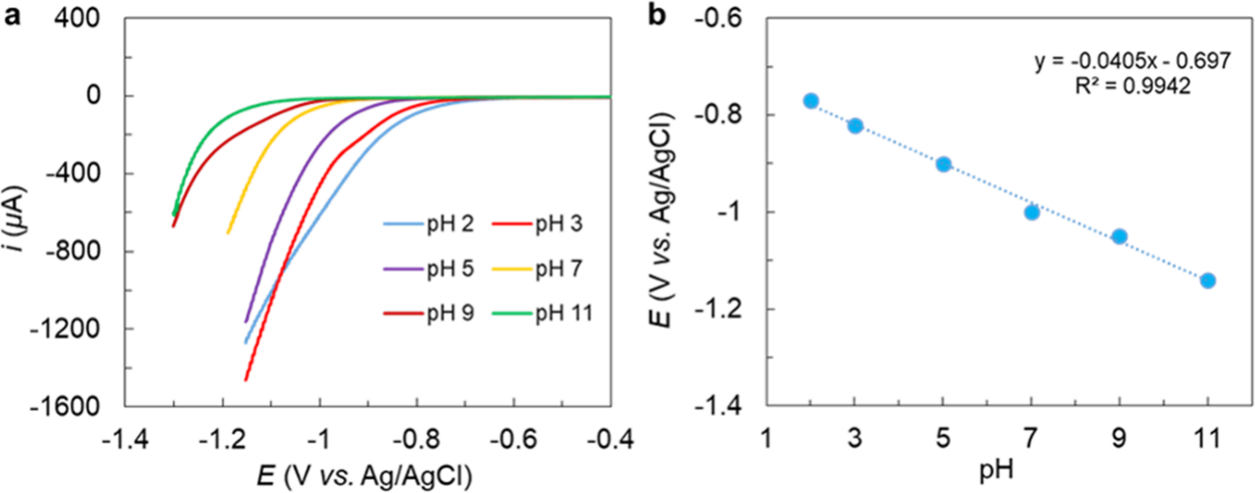
(a) Linear sweep voltammogram with **1** as a catalyst at various pH values in aqueous solutions and (b) corresponding *E* (onset) *vs* pH plot.

Given the structural similarity with LDHs, we studied the electrochemical water-splitting catalytic activity of **1**. In contrast to the insolubility of LDHs, compound **1** is readily soluble in water, and therefore, we have performed the catalytic studies for hydrogen evolution reaction (HER) with a 0.5 mM catalyst concentration at different pH in aqueous solutions. In the presence of the catalyst, the electrocatalytic response shows a lower overpotential for the HER than without the catalyst (Figure S18). The electrocatalytic response shows a gradual anodic shift with an increase of the current when the pH is lowered from pH 11 to 2, suggesting the catalyst to be active for HER across a wide range of pH values (Figure 5a). At pH 2, the catalyst-containing solution shows an onset potential of −0.77 V vs Ag/AgCl (−0.57 V vs NHE with a current density of 0.9 mA cm^–2^) and has an overpotential of ∼455 mV. The plot of the catalytic onset potential as a function of pH shows a slope of ∼ −40 mV/pH unit, suggesting a proton-coupled electron transfer mechanism in the potential determining step (Figure 5b). The Tafel slope is determined to be around 170 mV/dec and remains almost constant over the pH range (Figure S19). The impedance data shows a smaller semicircle in the presence of the catalyst, suggesting an easier charge transfer kinetics (Figure S20). Constant potential electrolysis revealed a Faradaic yield of ∼81% over 2 h of continuous operation (Figure S21) with a turnover frequency of 8.97 × 10^4^ μmol H_2_ h^–1^ mol^–1^ of the catalyst with no significant degradation (Figure S22). The catalyst is found to be homogeneous as there was no visible deposition on the glassy carbon surface, and the rinse test was negative (Figures S23 and S24). Moreover, the UV–Vis spectra of the catalyst in pure water and pH = 2 are identical, suggesting no significant pH-dependent change in the catalyst (Figure S25).

Dutta and co-workers have described that the electrocatalytic hydrogen evolution reaction of cobalt(III) complexes proceeds through the reduction to cobalt(II) complexes, which are further reduced to catalytically active cobalt(I) species under pH < 5.^28^ A close look at the crystal structure of compound **1** reveals that all of the Co^II^ centers are surrounded by six oxygen atoms (Co1 is connected to four hydroxides while Co4 is connected to two hydroxides), whereas the Co^III^ ions are coordinated to both O- and N-donor atoms. Therefore, it is difficult to predict which cobalt center is catalytically active. In an acidic medium, all the N-, O-, and hydroxido-groups have a chance to be protonated, leading to the cleavage of the metal–ligand bond to generate an open metal site with consequent reduction of the metal centers from cobalt(III) to cobalt(I) via cobalt(II) or directly from cobalt(II) to cobalt(I). Then, these catalytically active cobalt(I) centers reduce the H^+^ ions to H_2_.

To date, most of the electrocatalytic HER activity has been studied by using cobalt(II) or cobalt(III) complexes with very few studies performed on mixed-valence cobalt(II/III) complexes.^82^ As far as we know, none of these few studies report HER activity in the homogeneous phase, and therefore, this is the first report of electrocatalytic HER activity of a mixed-valence cobalt cluster in the homogeneous phase.

## 4 Conclusions

In summary, we have synthesized a bifunctional mixed-valence heptanuclear Co_3_^II^Co_4_^III^ complex showing field-induced SMM behavior and electrocatalytic HER activity. The Co_7_ cluster is formed under reflux conditions via subsequent partial dissociation of the compartmental Schiff base ligand along with the partial oxidation of cobalt(II) to cobalt(III). The Co_7_ cluster has a centrosymmetric wheel-shaped planar structure with hydroxide groups above and below the plane in a disposition similar to that of LDHs. The hydroxido-bridged linear Co_3_^II^ trimer within the cluster shows weak ferromagnetic Co^II^···Co^II^ interactions through the double hydroxido bridges, and the cluster shows a field-induced SMM behavior. A detailed literature survey shows that this is the first characterized SMM mixed-valence Co_7_ cluster.

Additionally, compound **1** shows electrocatalytic HER activity in the homogeneous phase over a wide range of pH (from 2 to 11). At pH = 2, it shows HER activity at moderate overpotential with significant Faradaic efficiency and TOF. Studies on such bifunctional materials with single-molecule magnet behavior and potential electrocatalytic activity will lead to the design of advanced materials for spin-controlled electrocatalysis.

## Supporting Information Available

Coordination bond lengths, bond angles, and hydrogen bond dimensions are given in Tables S1–S3. Crystal morphology, IR spectra of the ligand (H_5_L) and **1**, absorption spectra of the ligand and **1**, ^1^H NMR spectra of the ligand, XRPD of **1**, SEM micrographs of a microcrystalline sample of **1**, XPS of **1**, ORTEP diagram of **1**, coordination geometry of different metal centers, binding modes of the ligands, structural comparison of **1** with NiFe-LDH, different magnetic plots χ_m_ vs *T*, isothermal magnetization at different temperatures, and FC-ZFC and magnetic relaxation along with different electrochemical plots, such as linear sweep voltammetry, Tafel plots, constant potential electrolysis, absorption spectra before and after electrocatalysis, image of the electrode before and after electrocatalysis, Rinse test, and UV–Vis spectra of **1** before and after electrocatalysis, are presented in Figures S1–S25 within the Supporting Information file (PDF)
