# Supplementary material for: Heptanuclear Mixed-Valence Co4IIICo3II Molecular Wheel—A Molecular Analogue of Layered Double Hydroxides with Single-Molecule Magnet Behavior and Electrocatalytic Activity for Hydrogen Evolution Reactions
Source: Inorg Chem. 2024 Mar 25;63(14):6161–72. doi: 10.1021/acs.inorgchem.3c04065 (PMC11005049; doi:10.1021/acs.inorgchem.3c04065)
Supplement: Supplementary file 1 — ic3c04065_si_001.pdf [file ic3c04065_si_001.pdf]

Supporting Information

Heptanuclear Mixed Valence $Co^{III}_4Co^{II}_3$ Molecular Wheel – A Molecular Analogue of Layered Double Hydroxides with Single-Molecule Magnet Behavior and Electrocatalytic Activity for Hydrogen Evolution Reaction

Biplab Biswas,[1,2,†] Anjila I. Siddiqui,[3,†] Mithun Chandra Majee,[4] Swadhin Kumar Saha,[1] Biswajit Mondal,*[3] Rajat Saha,*[1,5] Carlos J. Gómez García*[5]

[1]*Department of Chemistry, Kazi Nazrul University, Asansol-713340, WB, India.*

[2]*Department of Chemistry, Hooghly Mohsin College, Chinsurah-712101, WB, India.*

[3]*Department of Chemistry, IIT Gandhinagar, Palaj, Gujarat,382355, India.*

[4]*Department of Chemistry, BB College, Asansol-713303, WB, India.*

[5]*Departamento de Química Inorgánica, Universidad de Valencia, 46100 Burjasot, Valencia, Spain.*

Corresponding authors: *carlos.gomez@uv.es (CJGG), rajatinorg1@gmail.com/rajat.saha@uv.es (RS), mondal.biswajit@iitgn.ac.in (BM)*

[†] Both have equal contributions.

Table of Contents	page
**Table S1**. Selected bond lengths (Å) in compound **1**	S3
**Table S2**. Selected bond angles (º) in compound **1**	S3
**Table S3**. Hydrogen bond dimensions in compound **1**	S4
**Figure S1.** Needle like single crystals of compound **1**	S4
**Figure S2**. IR Spectrum of the ligand H_5L^1	S5
**Figure S3.** IR Spectra of compound **1**	S5
**Figure S4.** ^{1}H-NMR spectra of the ligand H_5L^1	S6
**Figure S5**. Absorption spectra of the ligand H_5L^1 in methanol	S6
**Figure S6**. Absorption spectra of compound **1** in methanol	S7
**Figure S7.** Experimental and simulated XRPD patterns of compound **1**	S7
**Figure S8.** SEM micrographs showing the flake-like morphology of compound **1**	S8
**Figure S9.** XPS of compound **1** showing the presence of both Co^{2+} and Co^{3+} ions	S8
**Figure S10**. ORTEP view of the asymmetric unit of compound **1** with the labelling scheme of the main atoms.	S9
**Figure S11**. Coordination environment of the four different cobalt centers in complex **1**	S9
**Figure S12**. Different binding modes of the ligands $(H_2L^1)^{3-}$ (**a**) and $(HL^2)^-$ (**b**) in **1**.	S10
**Figure S13.** Structural comparison of complex **1** with NiFe-LDH.	S10
**Figure S14**. Thermal variation of the molar magnetic susceptibility for compound **1**	S11
**Figure S15**. Isothermal magnetizations at 1.9 K and 5.0 K for compound **1**	S11
**Figure S16**. Zero field cooled (ZFC) and field cooled (FC) molar magnetic susceptibility for compound **1** measured with 1 and 10 mT after cooling in zero field	S12
**Figure S17**. Field dependence of the relaxation time for compound **1** at 2.0 K	S12
**Figure S18**. Linear sweep voltammograms for HER without catalyst using blank glassy carbon electrode (black trace) and with 0.5 mM catalyst (red trace) at different pH in the range 2-11.	S13
**Figure S19**. Tafel plots at the kinetic region corresponding to Figure 5 for all pH values	S13
**Figure S20**. Impedance plot at pH = 2 and pH = 11	S14
**Figure S21.** Constant potential electrolysis. Experimental Conditions: 1 cm x 1 cm carbon cloth, Ag/AgCl and Pt wire were used as working, reference and counter electrode, respectively; pH 2; electrolysis potential = -1.2V	S14
**Figure S22**. UV-Vis spectra before and after the electrochemical experiment at pH = 2	S15
**Figure S23**. Image of a glassy carbon electrode before and after the rinse test at pH = 2	S15
**Figure S24**. Rinse test with 0.2 mM of catalyst.	S16
**Figure S25**. UV-VIS spectra of the catalyst in pure water and at pH = 2	S16

Table S1. Selected bond lengths (Å) in compound **1**.

Atoms	Length (Å)	Atoms	Length (Å)	Atoms	Length (Å)
Co1-O6	2.103(4)	Co2-O6	1.954(4)	Co3-O9	1.908(4)
Co1-O8	2.054(4)	Co2-O8	1.920(4)	Co3-N1	1.869(6)
Co1-O9	2.032(5)	Co2-N2	1.857(6)	Co4-O2	2.077(6)
Co1-O6*	2.103(4)	Co2-N3	1.953(6)	Co4-O3	2.128(5)
Co1-O8*	2.054(4)	Co3-O1	1.909(5)	Co4-O9	2.078(4)
Co1-O9*	2.032(5)	Co3-O3	1.899(5)	Co4-O4*	2.064(5)
Co2-O1	1.899(5)	Co3-O6	1.976(5)	Co4-O5*	2.093(4)
Co2-O5	1.892(5)	Co3-O7	1.924(5)	Co4-O8*	2.119(5)

* = 1-x, 1-y, -z

Table S2. Selected bond angles (º) in compound **1**.

Atoms	Angle (º)	Atoms	Angle (º)	Atoms	Angle (º)
O6-Co1-O8	79.55(15)	O5-Co2-O6	98.8(2)	O3-Co3-N1	86.4(2)
O6-Co1-O9	78.85(17)	O5-Co2-O8	84.62(19)	O6-Co3-O7	92.4(2)
O6-Co1-O6*	180.00	O5-Co2-N2	86.3(2)	O6-Co3-O9	85.09(18)
O6-Co1-O8*	100.45(15)	O5-Co2-N3	91.8(2)	O6-Co3-N1	174.5(2)
O6-Co1-O9*	101.15(17)	O6-Co2-O8	86.70(16)	O7-Co3-O9	177.4(3)
O8-Co1-O9	95.20(17)	O6-Co2-N2	174.7(2)	O7-Co3-N1	88.8(3)
O6*-Co1-O8	100.45(15)	O6-Co2-N3	85.9(2)	O9-Co3-N1	93.7(2)
O8-Co1-O8*	180.00	O8-Co2-N2	92.45(19)	O2-Co4-O3	89.0(2)
O8-Co1-O9*	84.80(17)	O8*-Co4-O9	82.04(17)	O2-Co4-O9	93.9(2)
O6*-Co1-O9	101.15(17)	O4*-Co4-O8*	92.5(2)	O2-Co4-O4*	92.9(2)
O8*-Co1-O9	84.80(17)	O8-Co2-N3	171.2(2)	O2-Co4-O5*	97.56(19)
O9-Co1-O9*	180.00	N2-Co2-N3	95.4(2)	O2-Co4-O8*	170.83(19)
O6*-Co1-O8*	79.55(15)	O1-Co3-O3	176.0(2)	O3-Co4-O9	75.02(17)
O6*-Co1-O9*	78.85(17)	O1-Co3-O6	79.2(2)	O3-Co4-O4*	95.93(19)
O8*-Co1-O9*	95.20(17)	O1-Co3-O7	88.4(2)	O3-Co4-O5*	171.31(19)
O1-Co2-O5	175.86(18)	O1-Co3-O9	91.71(18)	O3-Co4-O8*	97.86(17)
O1-Co2-O6	80.0(2)	O1-Co3-N1	95.4(2)	O4*-Co4-O9	168.57(19)
O1-Co2-O8	91.3(2)	O3-Co3-O6	98.9(2)	O5*-Co4-O9	98.70(16)
O1-Co2-N2	94.8(2)	O3-Co3-O7	95.2(2)	O4*-Co4-O5*	89.51(19)
O1-Co2-N3	92.1(3)	O3-Co3-O9	84.58(18)	O5*-Co4-O8*	75.07(17)

* = 1-x, 1-y, -z

Table S3. Hydrogen bond dimensions in compound **1**.

**D-H···A**	**D-H/(Å)**	**H···A/(Å)**	**D···A/(Å)**	**<D-H···A/(º)**	**Symmetry**
O4-H1O4···O1W	0.85	1.82	2.587(9)	149	
O7-H1O7···O13	0.85	1.86	2.688(13)	164	1-x, 1-y, -z
N3-H3A···O13	0.89	2.10	2.915(13)	152	x, y, 1+z
N3-H3B···O16	0.89	2.57	3.41(2)	157	x, y, 1+z
O8-H1O8···O10	0.93	1.85	2.759(12)	165	
O9-H1O9···O2W	0.92	1.80	2.655(8)	154	
O1W-H2W1···O3	0.85	1.98	2.816(9)	170	1+x, y, z
O2W-H1W2···O10	0.85	2.47	3.117(14)	133	
O2W-H1W2···O12	0.85	2.21	2.899(13)	138	
O3W-H1W3···O15	0.85	1.85	2.34(3)	116	
O3W-H2W3···O16	0.85	2.06	2.56(3)	118	
O3W-H2W3···O17	0.85	2.55	3.26(2)	141	
C8-H8···O17	0.93	2.44	3.356(16)	170	1-x, 1-y, -z
C11-H11A···O12	0.97	2.57	3.512(14)	163	1-x, -y, -z
C12-H12B···O14	0.97	2.39	3.317(13)	159	1-x, 1-y, -z
C14-H14B···O14	0.97	2.58	3.293(18)	131	2-x, 1-y, -z
C15-H15A···O16	0.97	2.60	3.56(2)	173	x, y, 1+z

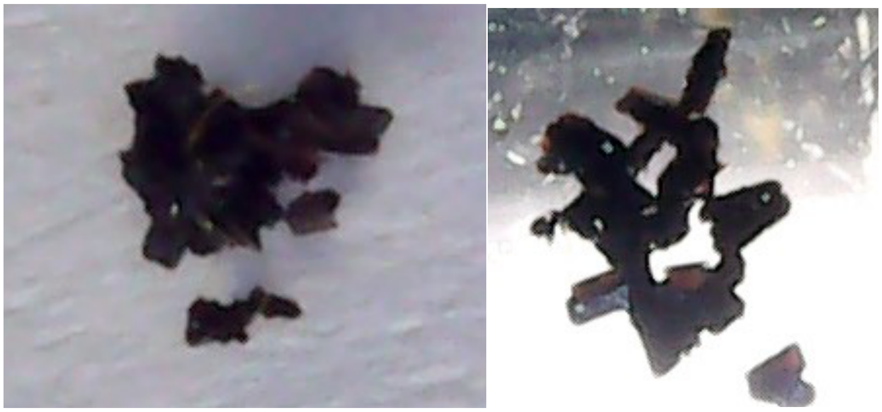

Figure S1. *Needle like single crystals of compound* ***1****.*

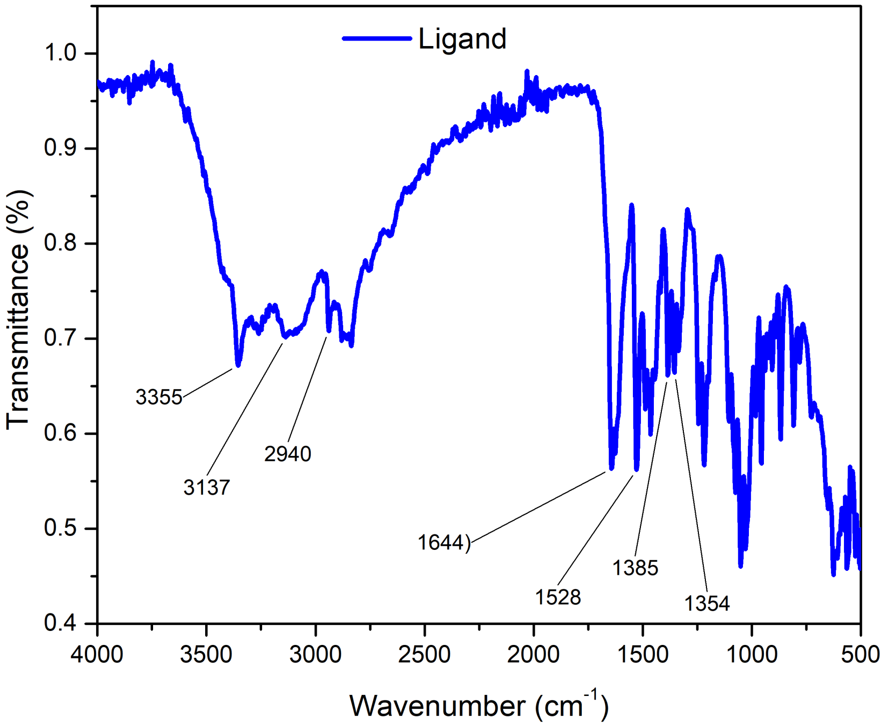

Figure S2. *IR Spectrum of the ligand H_5L^1.*

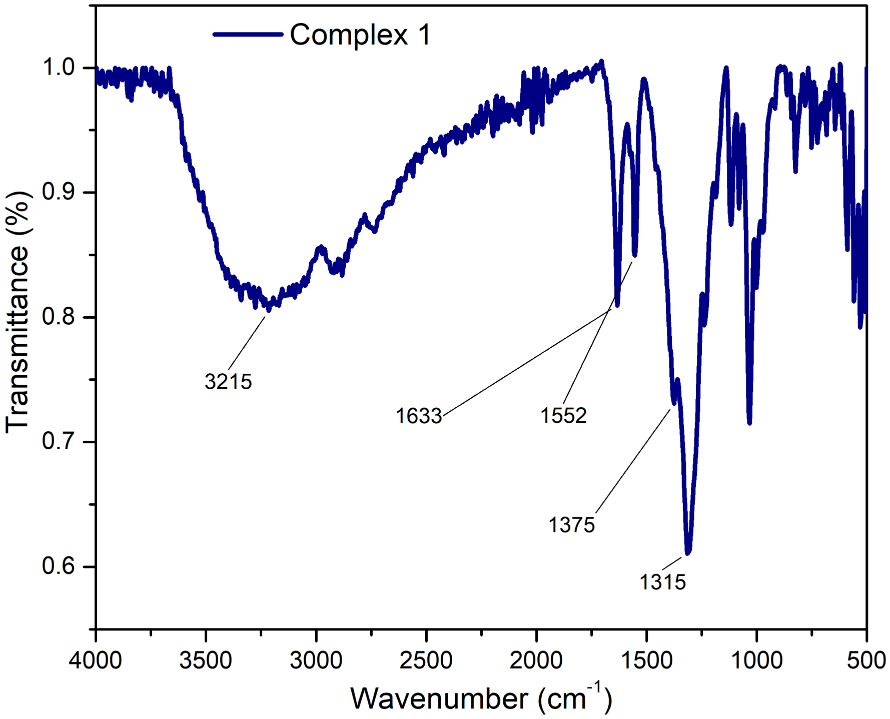

Figure S3. *IR Spectra of compound **1**.*

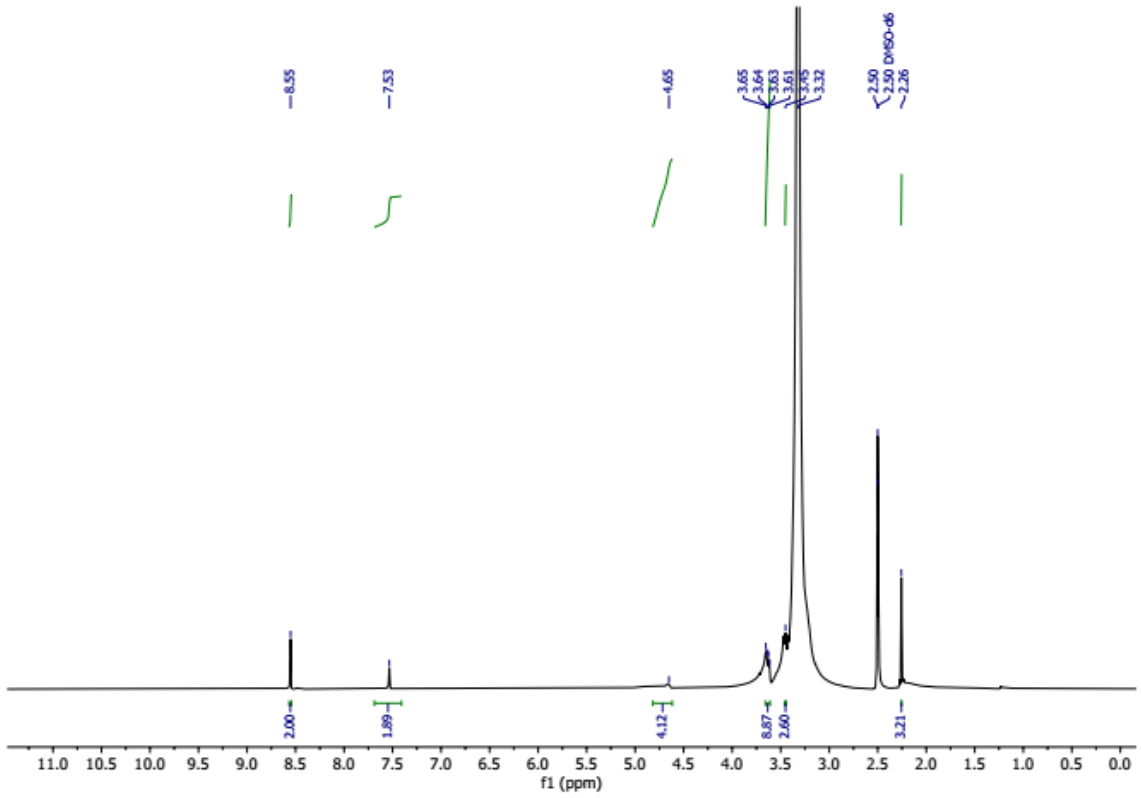

Figure S4: *^{1}H-NMR spectrum of the ligand H_5L^1.*

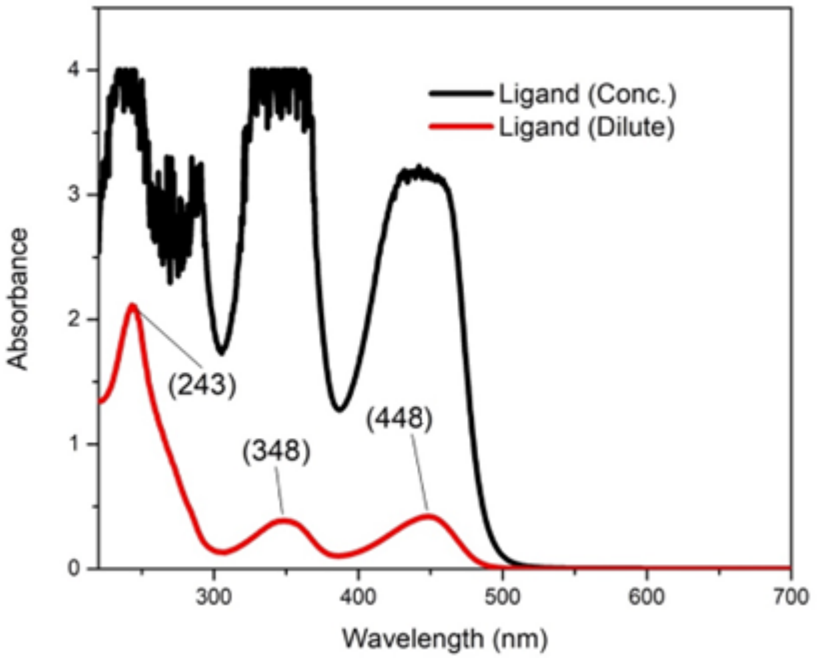

Figure S5*. Absorption spectra of the ligand H_5L^1 in methanol.*

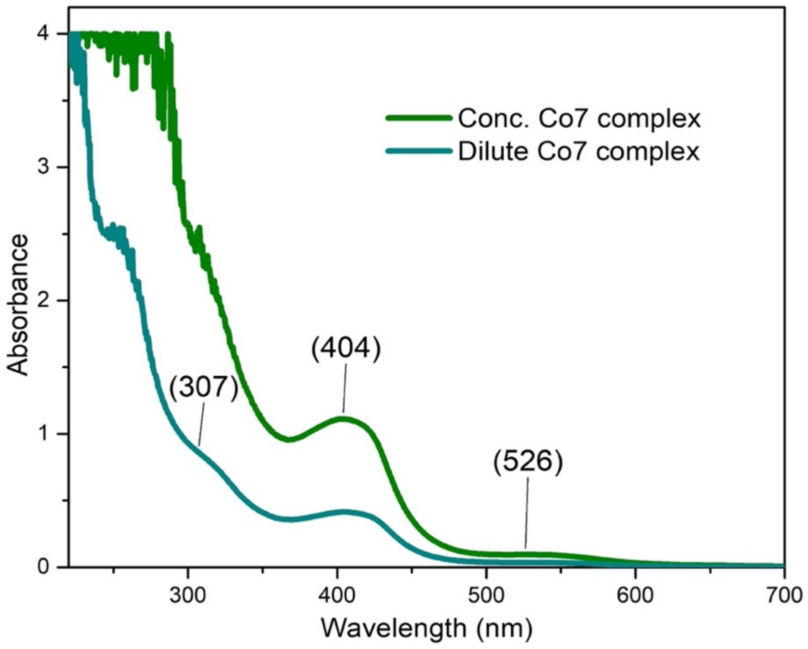

Figure S6*. Absorption spectra of compound* ***1*** *in methanol.*

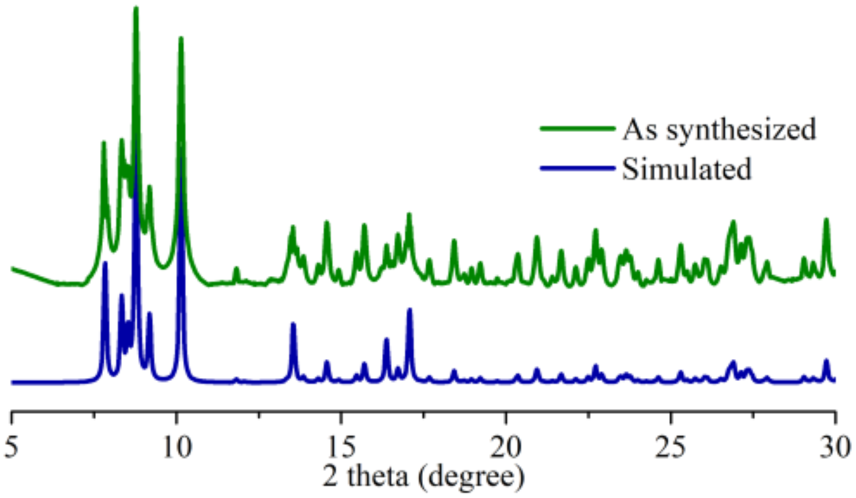

Figure S*7. Experimental and simulated XRPD patterns of compound* ***1****.*

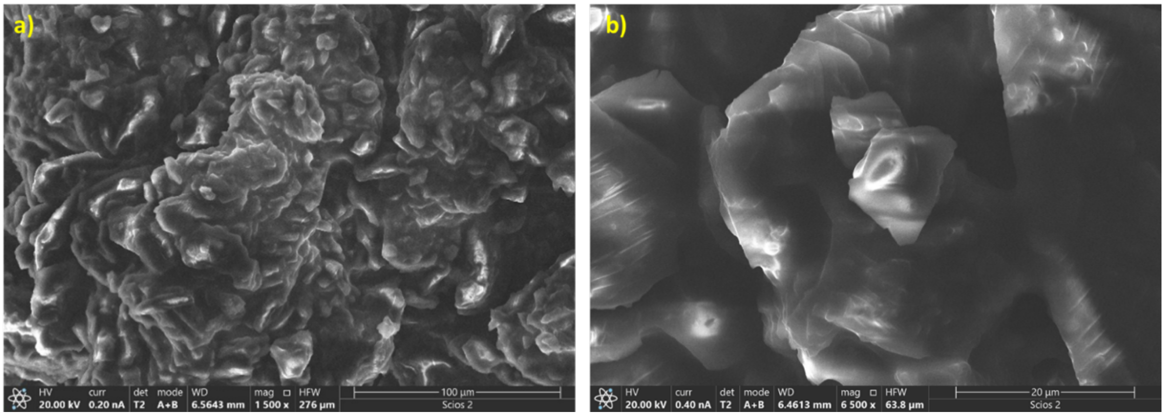

Figure S8. *SEM micrographs showing the flake-like morphology of compound **1**.*

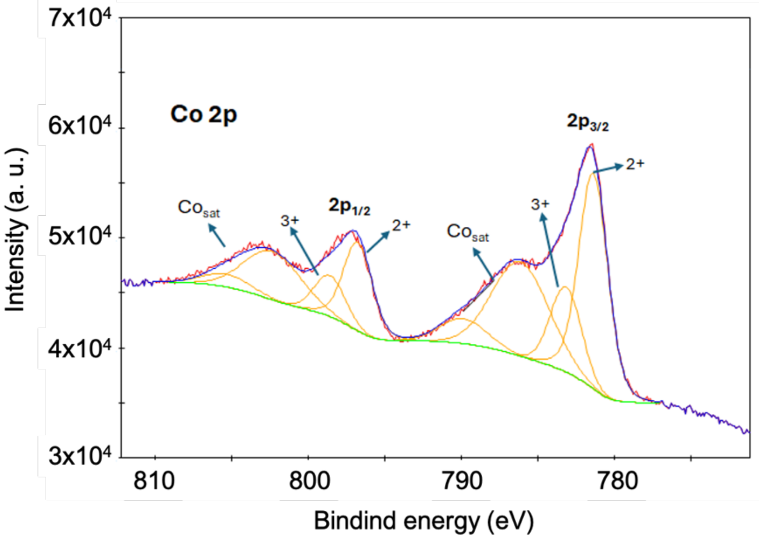

Figure S9. *XPS of compound **1** showing the presence of both Co^{2+} and Co^{3+} ions.*

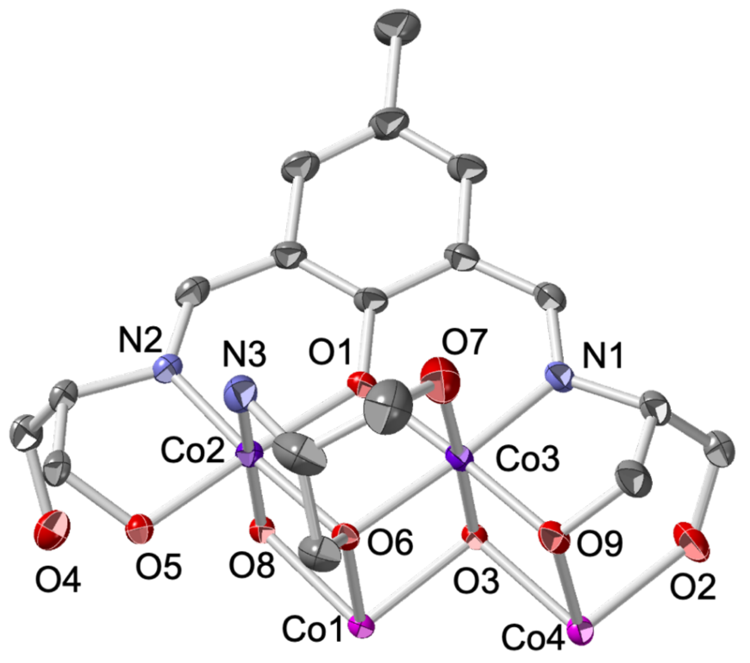

Figure S10. *ORTEP view of the asymmetric unit of compound* ***1*** *with the labelling scheme of the main atoms. H-atoms, the three* NO_3^- *anions and the three crystallization water molecules are omitted for clarity. Color code: C = grey, O = red,* Co^{II} *= pink,* Co^{III} *= violet and N = blue. Ellipsoids are drawn at 30 % probability.*

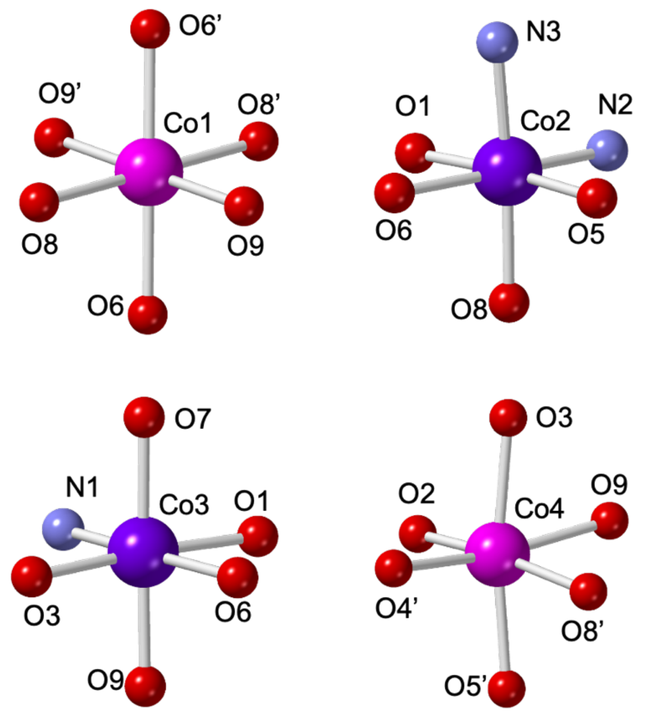

Figure S11. *Coordination environment of the four different cobalt centers in complex* ***1***.

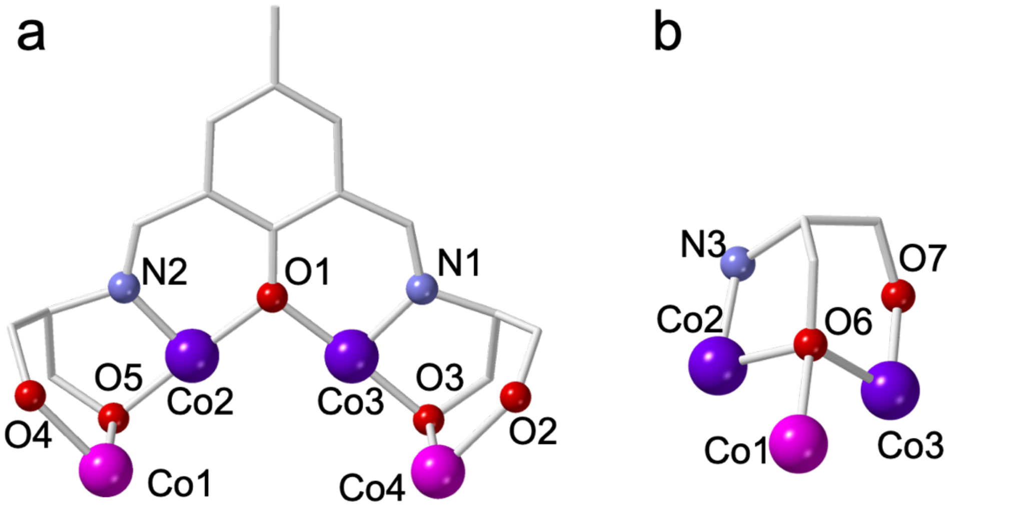

Figure S12. *Different binding modes of the ligands* $(H_2L^1)^{3-}$ (***a***) *and* $(HL^2)^-$ (***b***) *in* ***1***.

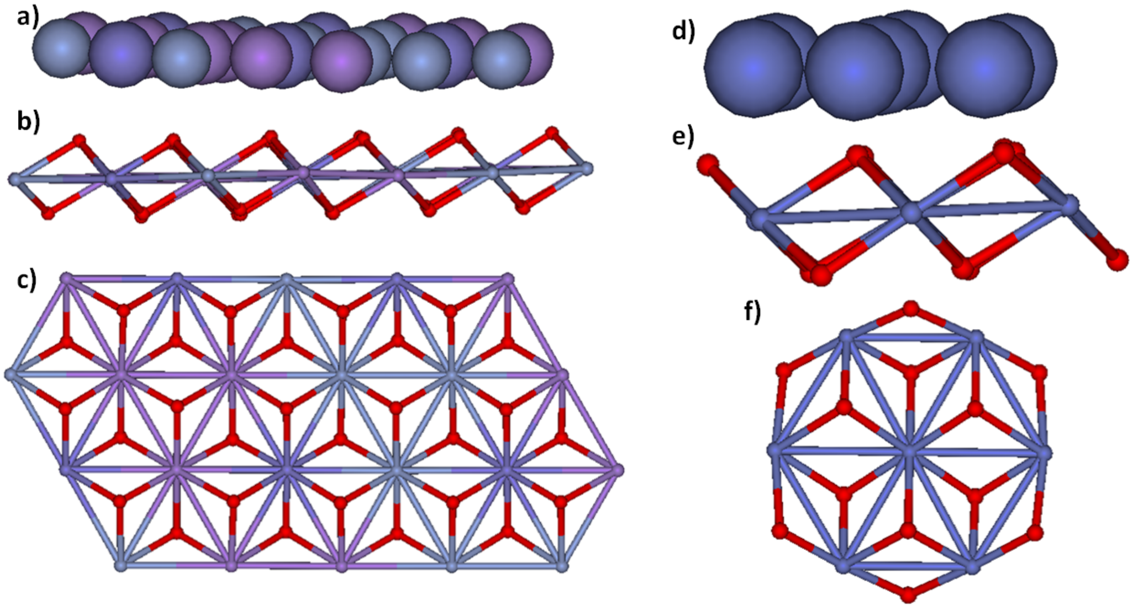

Figure S13. *Structural comparison of complex* ***1*** *with NiFe-LDH (JCPDS Card 40-0215).* (***a***) *Sphere model of the cations present in a layer of NiFe-LDH.* (***b***) *Side view of a layer of Ni-Fe-LDH.* (***c***) *Top view of a layer of Ni-Fe-LDH.* (***d***) *Sphere model of the cations present in a layer of compound* ***1***. (***e***) *Side view of a layer of compound* ***1***. (***f***) *Top view of the* Co_7 *cluster in compound* ***1***. *Color code: Coordinated hydroxides/oxides = red. Ni/Fe/Co = blue.*

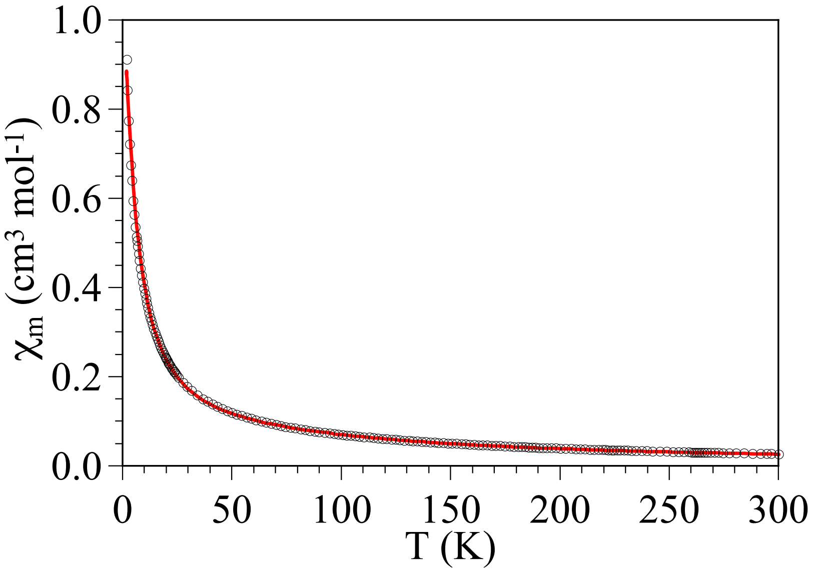

Figure S14. *Thermal variation of the molar magnetic susceptibility for compound* ***1***. *Solid line is the best fit to the model (see text).*

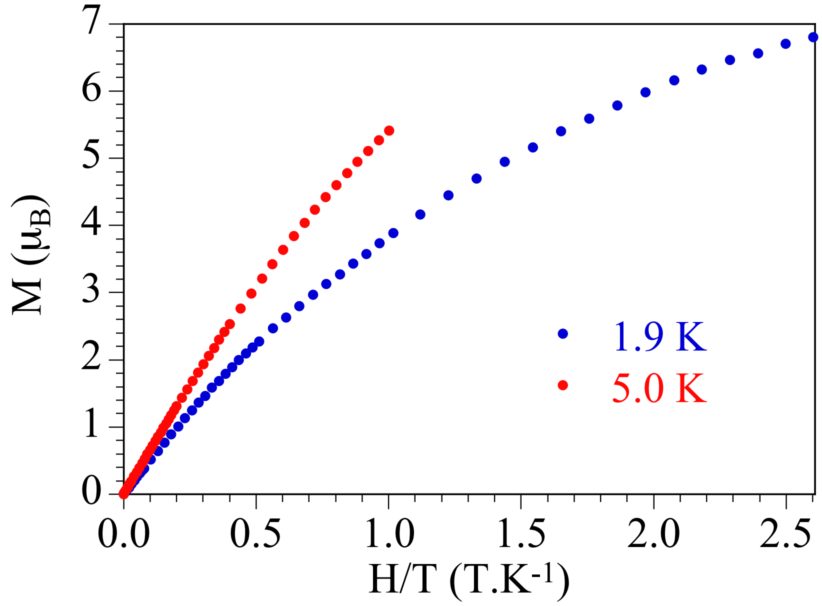

Figure S15. *Isothermal magnetizations at 1.9 K and 5.0 K for compound* ***1***.

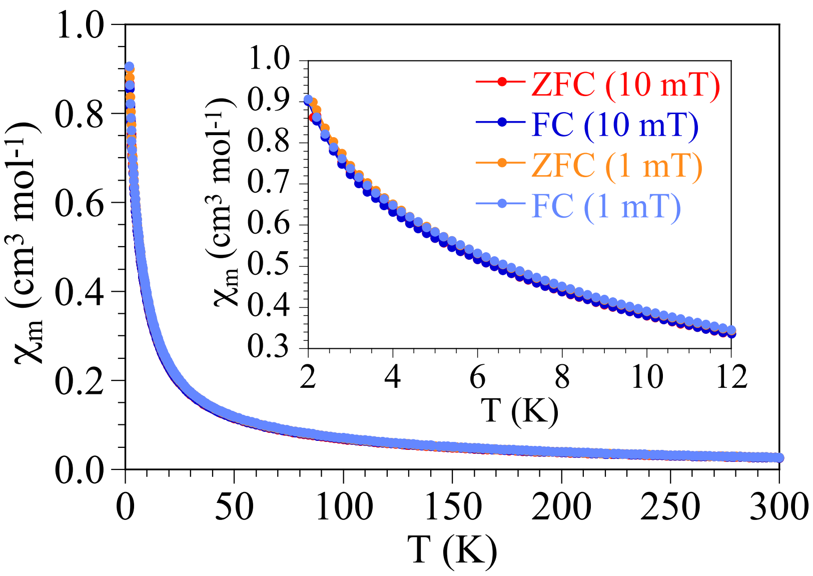

***Figure S16**. Zero field cooled (ZFC) and field cooled (FC) molar magnetic susceptibility for compound **1** measured with 1 and 10 mT after cooling in zero field.*

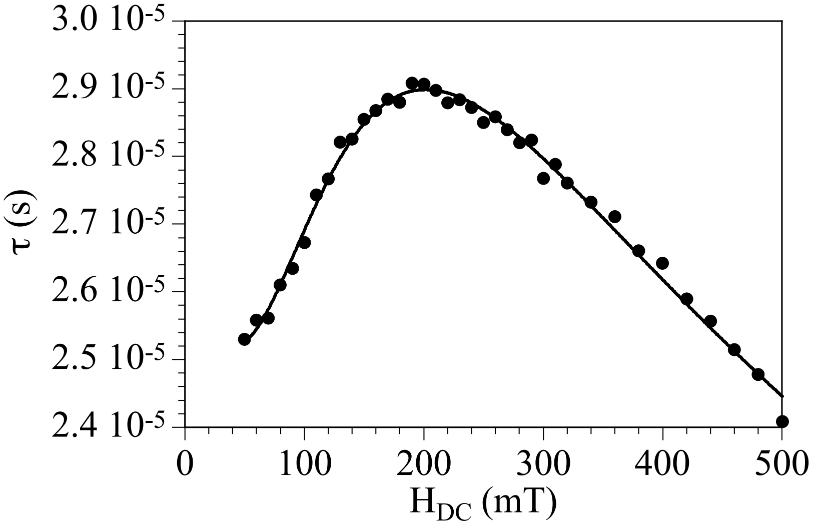

***Figure S17**. Field dependence of the relaxation time for compound **1** at 2.0 K. Solid line is the best fit to equation (1).*

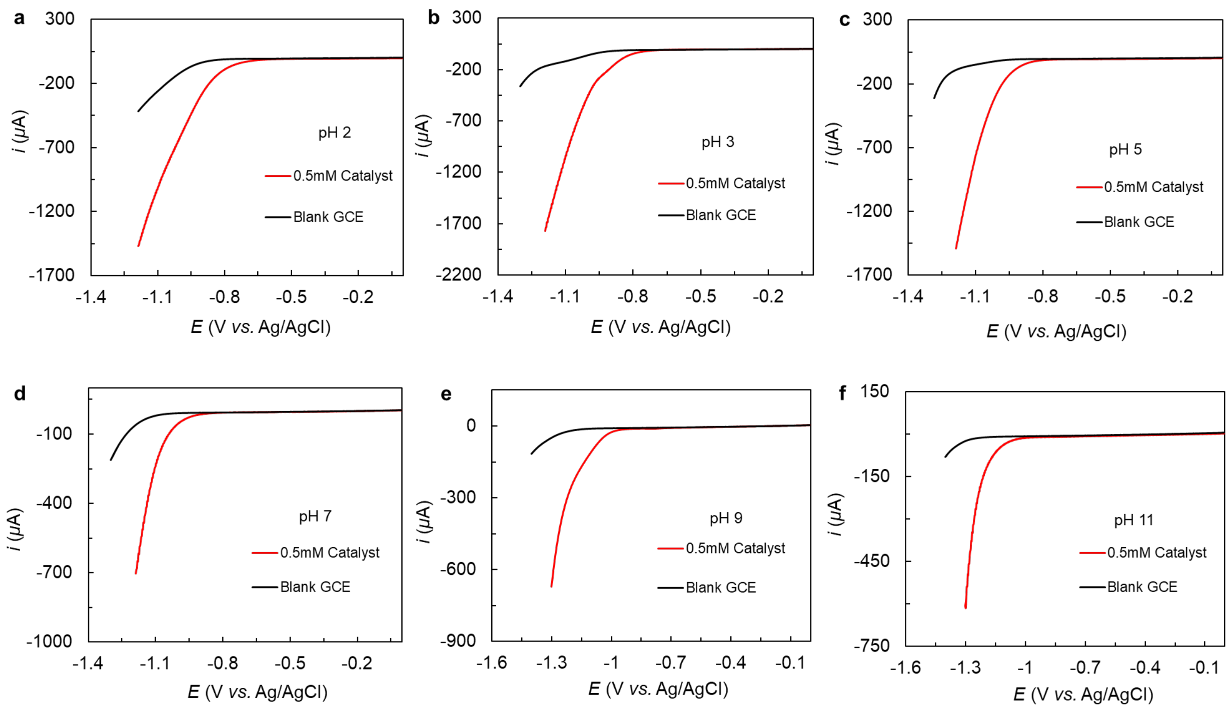

Figure S18. *Linear sweep voltammograms for HER without catalyst using blank glassy carbon electrode (black trace) and with 0.5 mM catalyst (red trace) at different pH in the range 2-11* ***(a)-(f)***.

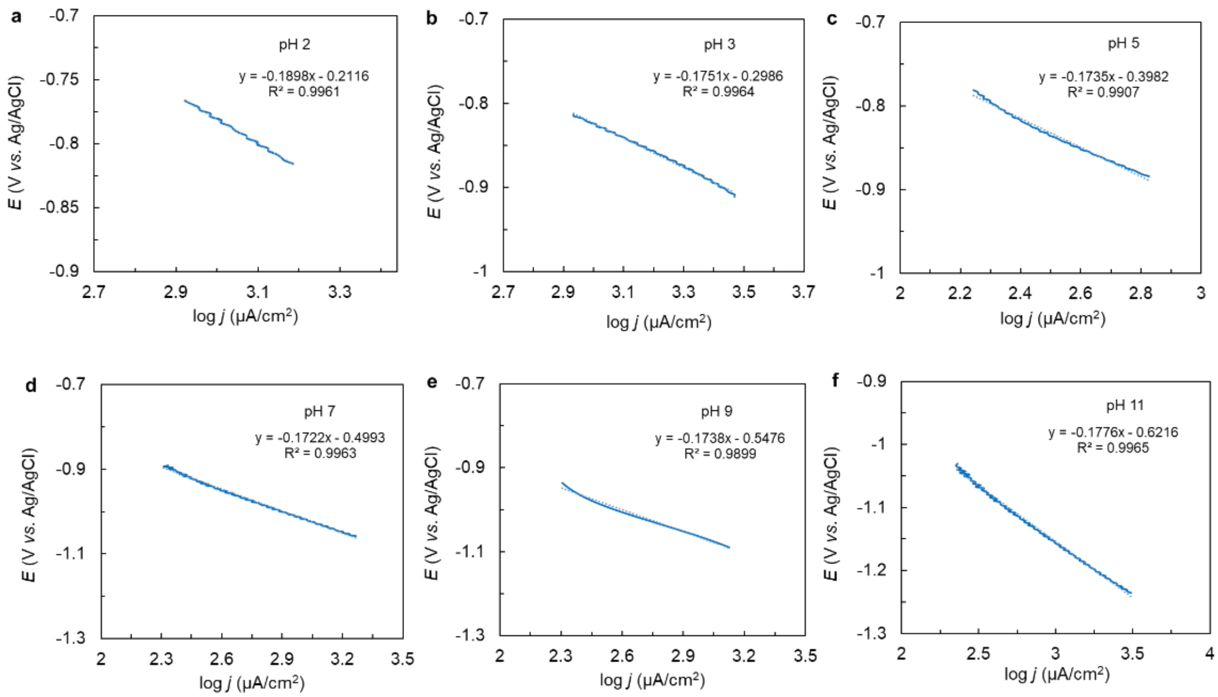

Figure S19. ***(a)-(f)*** *Tafel plots at the kinetic region corresponding to Figure 5 for all pH values.*

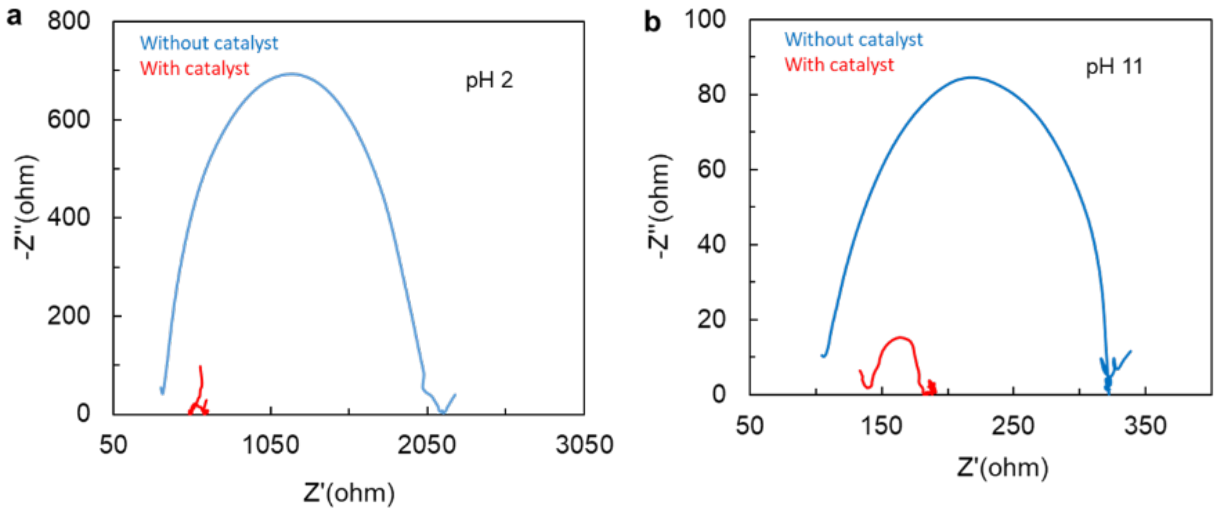

Figure S20. *Impedance plots at pH = 2 (**a**) and pH = 11 (**b**).*

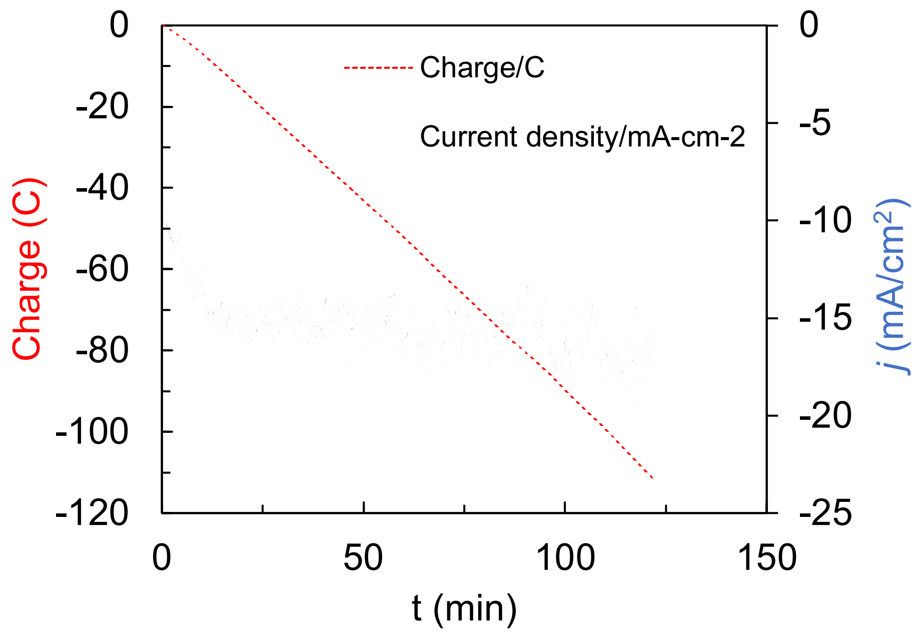

Figure S21. *Constant potential electrolysis. Experimental Conditions: 1 cm x 1 cm carbon cloth, Ag/AgCl and Pt wire were used as working, reference and counter electrode, respectively; pH = 2; electrolysis potential = -1.2 V.*

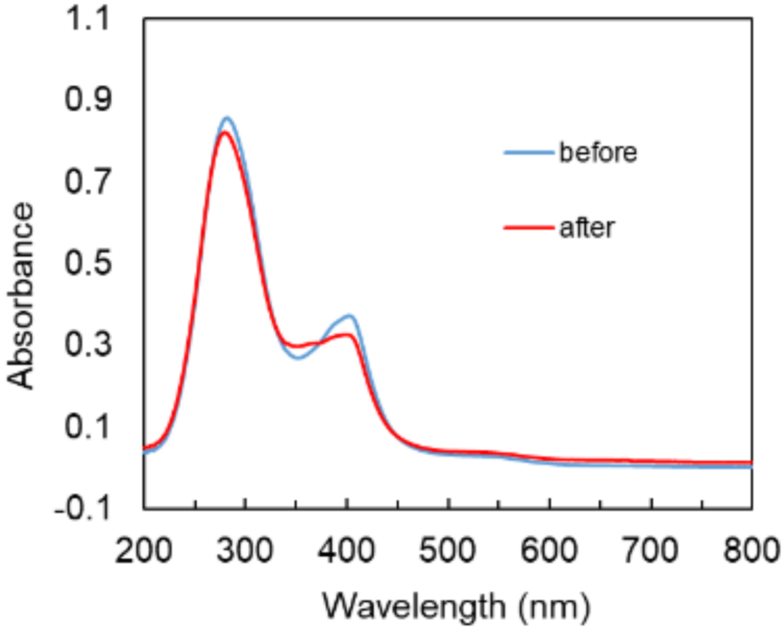

Figure S22. *UV-Vis spectra before and after the electrochemical experiment at pH = 2.*

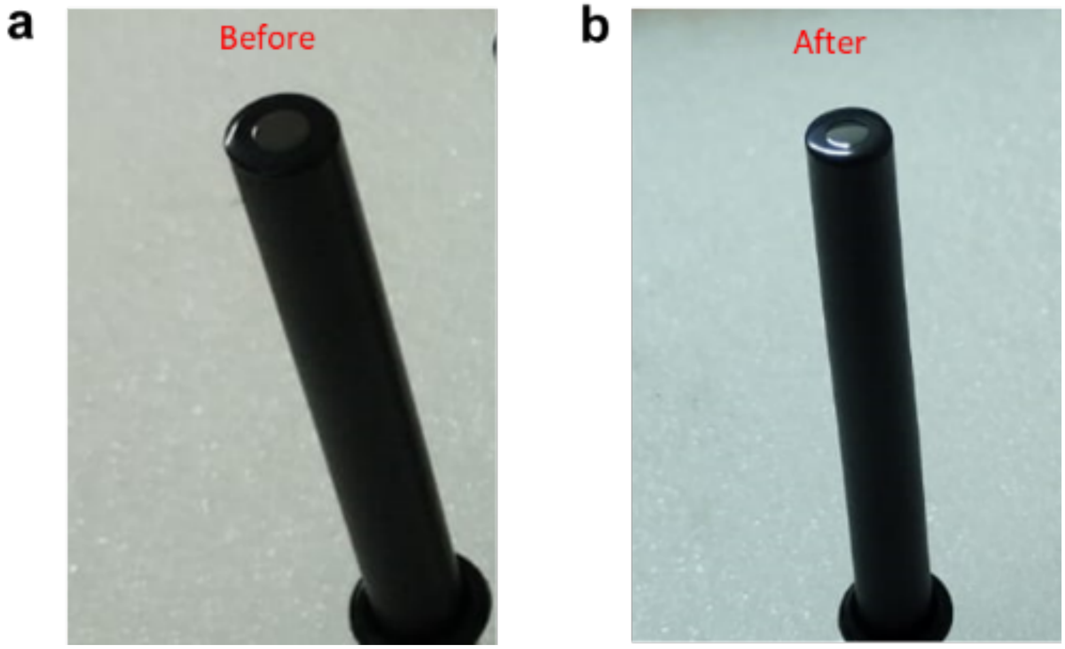

Figure S23. *Image of a glassy carbon electrode before (**a**) and after (**b**) the rinse test at pH = 2.*

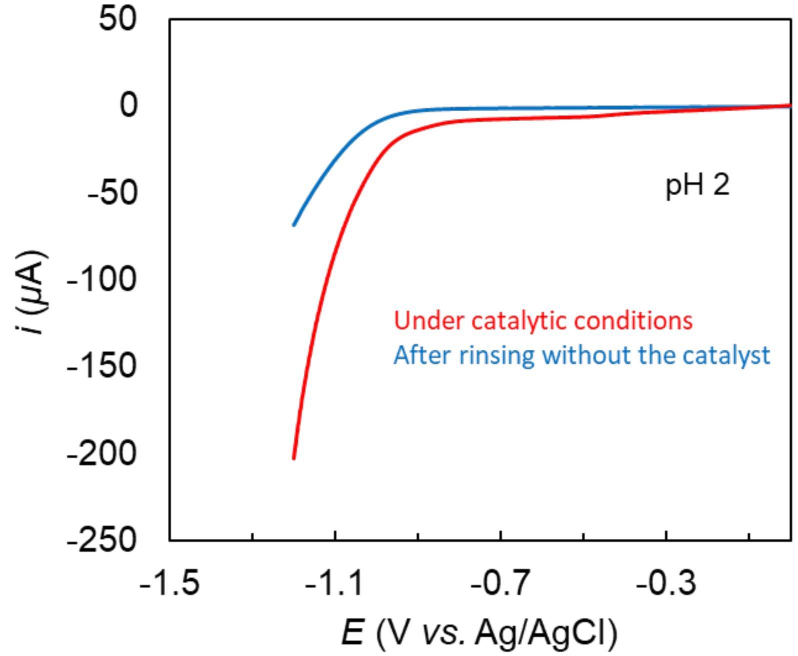

Figure S24. *Rinse test with 0.2 mM of catalyst. In this test, the same working electrode after the catalysis is used to perform CV experiments in a catalyst-free solution at the same pH without mechanical polishing of the electrode.*

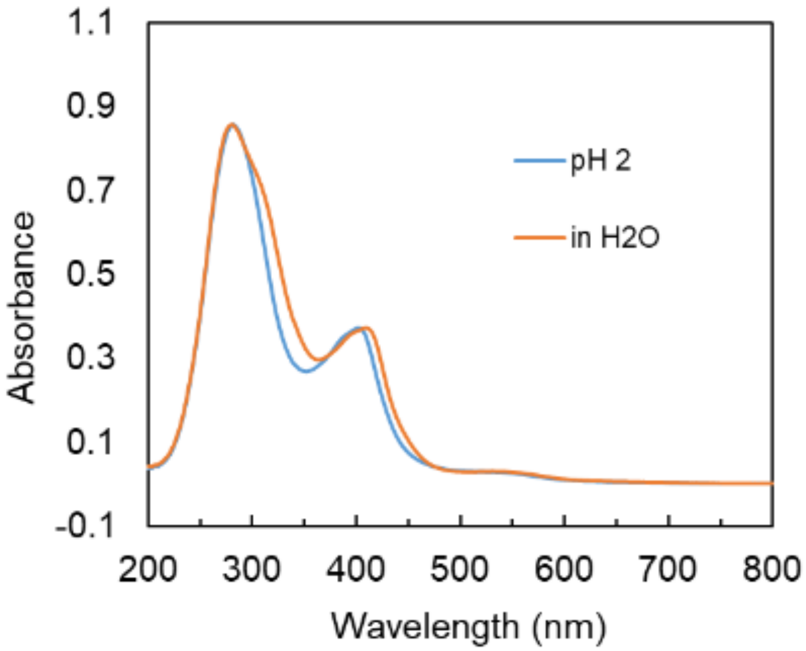

Figure S25. *UV-VIS spectra of the catalyst in pure water and at pH = 2.*
